# Cirbp-PSD95 axis protects against hypobaric hypoxia-induced aberrant morphology of hippocampal dendritic spines and cognitive deficits

**DOI:** 10.1186/s13041-021-00827-1

**Published:** 2021-08-21

**Authors:** Yang Zhou, Huanyu Lu, Ying Liu, Zaihua Zhao, Qian Zhang, Chong Xue, Yuankang Zou, Zipeng Cao, Wenjing Luo

**Affiliations:** Department of Occupational and Environmental Health and the Ministry of Education Key Lab of Hazard Assessment and Control in Special Operational Environment, School of Public Health, Fourth Military Medical University, Xi’an, China

**Keywords:** Hypobaric hypoxia, High altitude environment, Cognitive impairment, Dendritic spine morphology, Cold-inducible RNA-binding protein, PSD95

## Abstract

Hypobaric hypoxia (HH) is a typical characteristic of high altitude environment and causes a spectrum of pathophysiological effects, including headaches, gliovascular dysfunction and cognitive retardation. Here, we sought to understand the mechanisms underlying cognitive deficits under HH exposure. Our results showed that hypobaric hypoxia exposure impaired cognitive function and suppressed dendritic spine density accompanied with increased neck length in both basal and apical hippocampal CA1 region neurons in mice. The expression of PSD95, a vital synaptic scaffolding molecule, is down-regulated by hypobaric hypoxia exposure and post-transcriptionally regulated by cold-inducible RNA-binding protein (Cirbp) through 3′-UTR region binding. PSD95 expressing alleviates hypoxia-induced dendritic spine morphology changes of hippocampal neurons and memory deterioration. Moreover, overexpressed Cirbp in hippocampus rescues HH-induced abnormal expression of PSD95 and attenuates hypoxia-induced dendritic spine injury and cognitive retardation. Thus, our findings reveal a novel mechanism that Cirbp-PSD-95 axis appears to play an essential role in HH-induced cognitive dysfunction in mice.

**Supplementary Information:**

The online version contains supplementary material available at 10.1186/s13041-021-00827-1.

## Introduction

Millions of people permanently reside in high altitude environment, which cover about one-fifth of the earth’s surface, and many have been there for generations [1]. In recent years, migration to high altitude regions has been an increasingly common activity, including travelers, mountaineers, miners and skiers. Hypobaric hypoxia is the most significant climatic characteristic of high altitude environment. For humans ascending to altitude, the lower partial pressure of oxygen leads to a reduction in the oxygen content of arterial blood and thence to tissue hypoxia [2, 3]. Researchers have reported that humans may suffer from cognitive impairment in high altitude exposure [4]. We also demonstrated that executive function characterized by working memory and psychomotor function is impaired in hypobaric hypoxia environment [5]. However, the underlying mechanisms of hypobaric hypoxia on cognitive deficits remain poorly understood.

Dendrites and dendritic spines are dynamic structures with pivotal roles in excitatory synapses in the mammalian neocortex and have been recognized as the locus underlying long-term synaptic plasticity, which is related to cognitive processes such as learning and memory [6]. Abnormal dendritic spine plasticity may contribute to neurological disorders. Whether and how long-time physiological hypoxia leads to dendritic spine deficits has yet to be determined. The protein PSD95, a membrane-associated guanylate kinase, is the major scaffolding protein in the excitatory postsynaptic density (PSD) and a potent regulator of synaptic strength [7]. PSD95 expression is increased to maximum levels in the adult, when spine stability and synaptic maturity are at their peak, and PSD95 overexpression leads to an increase in spine density as well as average spine size [8]. Thus, here we set to determine whether PSD95 is a potential therapeutic target for alleviating hypobaric hypoxia induced cognition impairment.

Cold-inducible RNA-binding protein (Cirbp) has been initially identified as a member of cold shock proteins and is expressed in a variety of mammalian cells, participating in various biological functions [9]. Endogenous and environmental stressors, including hypoxia have been shown to regulate the expression of Cirbp [10]. Our previous published work has shown that Cirbp sustained the proliferation of neural stem cells under hypoxic exposure, and the level of Cirbp was repressed under hypoxia conditioning [11]. However, it remains uncertain whether Cirbp can regulate the dendritic spine morphology, affecting hypoxia-induced cognitive dysfunction. Therefore, we investigated the role of Cirbp-PSD95 axis in hypoxia-induced dendritic spines plasticity abnormality and cognition impairment.

## Results

### Hypobaric hypoxia impaired cognitive function and dendritic spines morphology

**Fig. 1:**
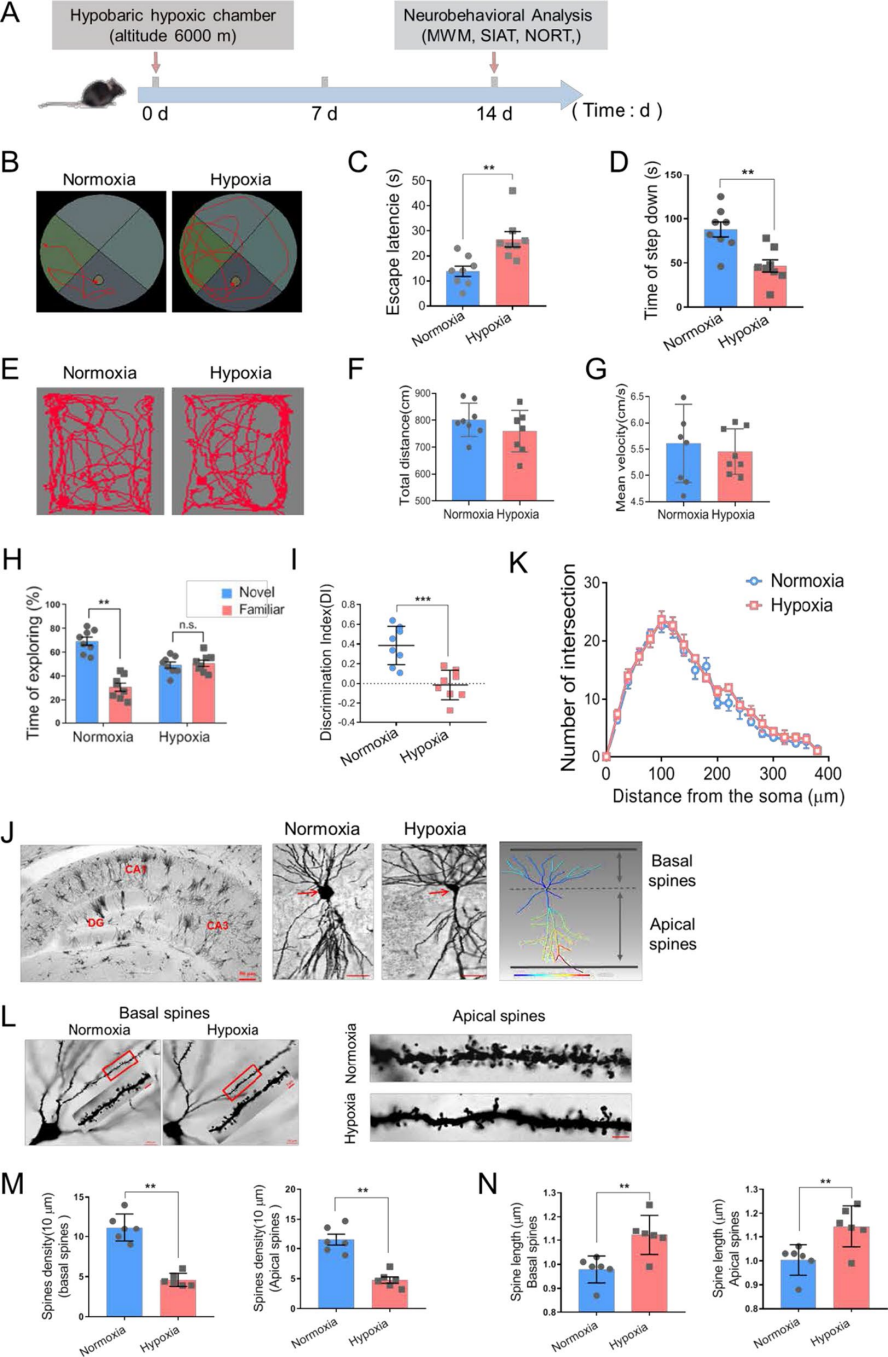
Hypobaric hypoxic exposure impaired memory ability and spine morphology. **A** The scheme of the experimental procedure. **B**, **C** Representative locomotion tracking plots (**B**) and the escape latency (**C**) of mice in MWM test (n = 8, Student’s *t*-test, ± SEM). **D** The latency time prior to descent from the platform in SIAT test (n = 8, Student’s *t*-test, ± SEM). **E**–**I** Representative locomotion tracking plots (**E**), the total distance (**F**), mean velocity (**G**), exploring time on new objects (**H**) and discriminate index (**I**) of mice in NORT test following normoxia or hypoxia exposure (n = 8, Student’s *t*-test, ± SEM). **J** Left: Representative Golgi staining of mice hippocampus. DG, dentate gyrus, scale bar = 50 μm. Middle: Golgi staining of CA1 pyramidal neurons, scale bar = 20 μm. Right: Imaris reconstruction of CA1 pyramidal neurons. **K** Number of dendritic intersections of reconstructed pyramidal neurons by Sholl analysis (three mice per group, Student’s *t*-test, ± SEM). **L** Left: Representative Golgi staining of basal dendritic spines in CA1, scale bar = 20 μm (Red frame indicated target area, scale bar = 5 μm). Right: Representative morphology of apical dendritic spines in CA1, scale bar = 5 μm. **M** Quantification of basal and apical dendritic spines density and columns represent number of spines per 10 μm (6 neurons/3 mice per group, Student’s *t*-test, ± SEM). **N** Quantification of basal and apical neck length (6 neurons/3 mice per group, Student’s *t*-test, ± SEM). *n.s.* no significant, *p < 0.05, **p < 0.01, ***p < 0.001

To assess the effect of hypobaric hypoxia on cognitive function of mice, we performed morris water maze (MWM), step-down inhibitory avoidance test (SIAT) and novel object recognition test (NORT) (Fig. 1A). MWM analysis is to detect the spatial reference memory ability. In the hidden platform trail, animals exposed to hypoxia exhibited an impaired ability to find the hidden platform, indicated by increased escape latency compared with animals in normoxic group shown in Fig. 1B, C (13.88 ± 2.15 for Normoxia vs. 26.63 ± 3.16 for Hypoxia, n = 8 per group, *p* = 0.004 Student’s *t*-test, Additional file 1). The SIAT analysis was employed to evaluate the working memory capacity. After 14 days, the latency for the mice in hypoxic group to step down the platform was significantly shortened compared to that in the normoxic group (87.81 ± 8.48 for Normoxia vs. 46.65 ± 6.99 for Hypoxia, n = 8 per group, *p* = 0.002 Student’s *t*-test, Fig. 1D), indicating that hypobaric hypoxia exposure hindered working memory in mice. In NORT analysis, which tests episodic memory capacity, the hypoxia exposed mice spent more time exploring a novel object than the normoxia exposure, specifying that hypobaric hypoxia exposure leads to aggravated episodic memory impairment under this condition (discrimination index: 0.386 ± 0.069 for Normoxia vs. − 0.015 ± 0.053 for Hypoxia, n = 8 per group, *p* < 0.001 Student’s *t*-test, Fig. 1E–G, Additional file 1). However, there was no difference in the total distance and the mean speed between the mice in the two groups, identifying that hypoxia did not affect the general locomotor activity (Fig. 1H, I, Additional file 1). These findings suggest that hypobaric hypoxia exposure leads to memory deterioration in mice, which is consistent with the existing studies [12–14].

Dendritic spines represent primary postsynaptic sites of excitatory synaptic inputs to pyramidal neurons, and studying densities and morphology of these structures provides invaluable information on morphofunctional characteristics of cognitive ability [15, 16]. Next, the morphology of dendritic spines remodeling was examined, showing there was no difference in the intersection number of the dendritic arborizations between two groups (Fig. 1J, K). Hypobaric hypoxia caused a significant reduction in overall dendritic spine density of both basal and apical dendrites of pyramidal neurons in murine hippocampus (basal spines density: 11.17 ± 1.72 for Normoxia vs. 4.77 ± 0.53 for Hypoxia, n = 6 per group, *p* = 0.004 Student’s *t*-test; apical spines density: 11.50 ± 0.91 for Normoxia vs. 4.60 ± 0.81 for Hypoxia, n = 6 per group, *p* = 0.003 Student’s *t*-test; Fig. 1L, M, Additional file 1). The neck length of both basal and apical dendritic protrusions increased in hippocampal pyramidal neurons (basal spines neck length: 0.978 ± 0.023 for Normoxia vs. 1.123 ± 0.034 for Hypoxia, n = 6 per group, *p* = 0.005 Student’s *t*-test; apical spines neck length: 1.003 ± 0.026 for Normoxia vs. 1.145 ± 0.035 for Hypoxia, n = 6 per group, *p* = 0.009 Student’s *t*-test; Fig. 1N, Additional file 1). Therefore, hypobaric hypoxic exposure induced abnormal morphology resembling of dendritic protrusions which may underlies impaired memory ability.

### PSD95 alleviates hypobaric hypoxia-induced aberrant morphology of dendritic spines and memory deficits

**Fig. 2:**
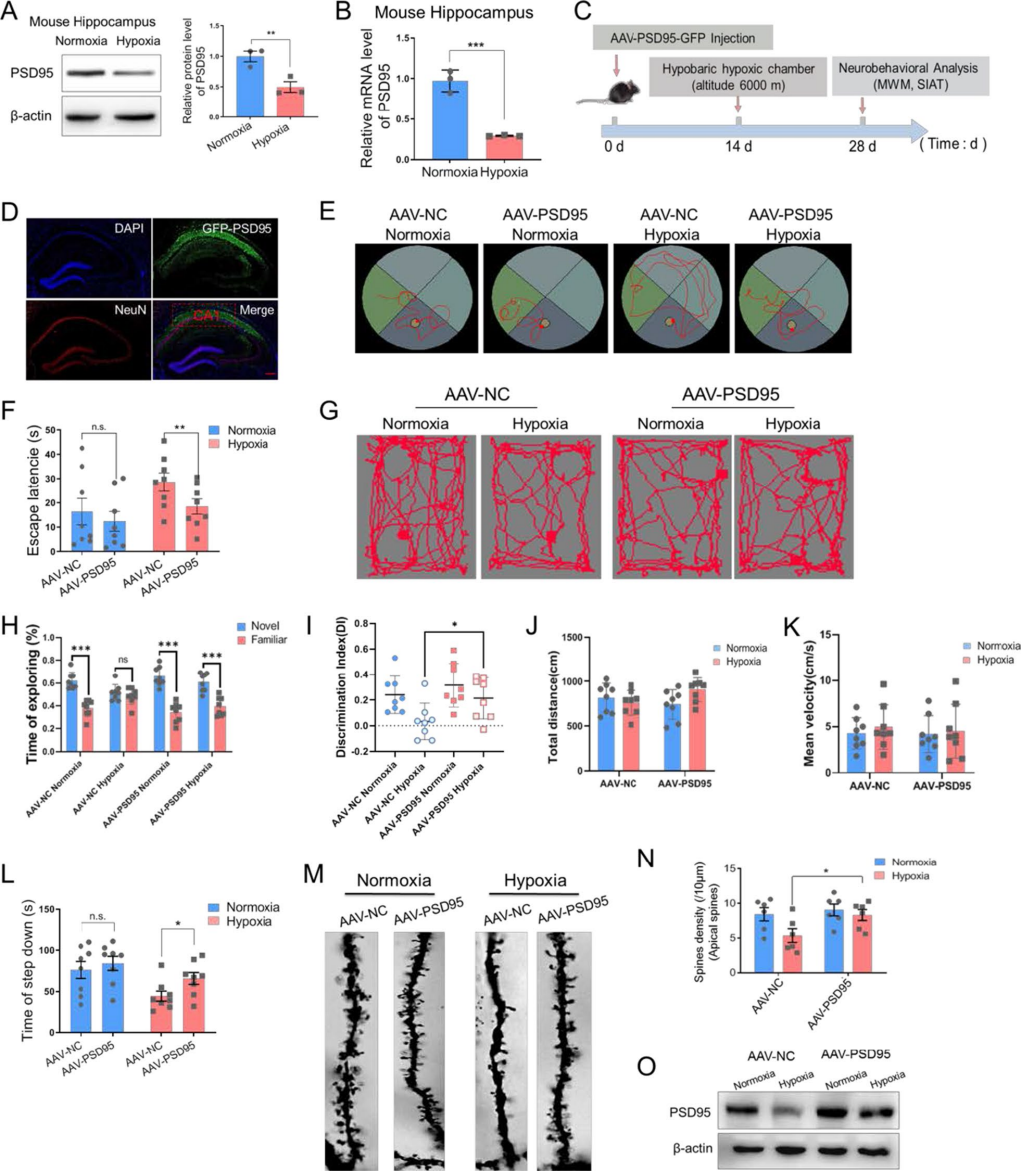
Hypobaric hypoxia down-regulated PSD95 protein level in mice hippocampus and ectopic expression of PSD95 restored memory function and the spine phenotype caused by hypoxic exposure. **A** Representative immunoblotting and the quantification analysis of PSD95 (n = 3 biological replicates, Student’s *t*-test, ± SEM). **B** The relative mRNA levels of *PSD95* in hippocampus following normoxia or hypoxia exposure (n = 3 biological replicates, Student’s *t*-test, ± SEM). **C** Schematic representation of the experimental setup. **D** The fluorescence image of the hippocampus CA1 after stereotacitic injection showing the autofluorescence of AAV-PSD95 (green) and DAPI (blue) and the immunofluorescence of NeuN (red), scale bar = 50 μm. **E**, **F** Representative tracking plots (**E**) and the escape latency (**F**) of mice under indicated treatment in MWM test (n = 8, two-way ANOVA, ± SEM). **G**–**K** Representative locomotion tracking plots (**G**), exploring time on new objects (**H**), discriminate index (**I**), total distance (**J**) and mean velocity (**K**) of mice under indicated treatment in NORT test (n = 8, two-way ANOVA, ± SEM). **L** The latency time of mice under indicated treatment in SIAT test (n = 8, two-way ANOVA, ± SEM). **M**, **N** Representative Golgi staining morphology (**M**, scale bar = 5 μm) and quantitative analysis of density (**N**) of apical spines in hippocampus CA1 neurons of mice under indicated treatment (6 neurons/3 mice per group, two-way ANOVA, ± SEM). **O** Representative immunoblotting of PSD95 (n = 3 biological replicates). *n.s.* no significant, *p < 0.05, **p < 0.01

To explore putative mechanism of the effect of hypoxic exposure on memory and dendritic spines, Western blot analysis demonstrated decreased PSD95 protein levels in hippocampus under hypoxic status (n = 3 biological replicates, *p* = 0.009 Student’s *t*-test, Fig. 2A, Additional file 1, 2). RT-PCR analysis revealed that the mRNA level of *PSD95* was down-regulated (n = 3 biological replicates, *p* < 0.001 Student’s *t*-test, Fig. 2B, Additional file 1, 2). To determine whether the observed decrease of PSD95 expression was associated with the hypoxia-induced memory deficits and dendritic spine abnormalities, we injected AAV-PSD95 into hippocampal CA1 region of mice (Fig. 2C, D) and exogenous PSD95 protein was expressed (Fig. 2O). MWM analysis revealed that the mice in AAV-PSD95 hypoxia group had reduced latency than AAV-NC hypoxia mice (significant difference between AAV-NC hypoxia vs. AAV-PSD95 hypoxia, n = 8, *p* = 0.002 two-way ANOVA, Fig. 2E, F, Additional file 1). In NORT analysis, PSD95 overexpression in hippocampal region significantly increased the discrimination index of hypoxia exposed mice when compared to the mice in the AAV-NC + hypoxia group (significant difference of discrimination index between AAV-NC hypoxia vs. AAV-PSD95 hypoxia, n = 8, *p* = 0.032 two-way ANOVA, Fig. 2G–I, Additional file 1). There was no obvious discrepancy of total distance and mean velocity between all groups (Fig. 2J, K, Additional file 1). Increased PSD95 also prolonged the platform leaving time after hypoxia exposure in SIAT experiment (significant difference between AAV-NC hypoxia vs. AAV-PSD95 hypoxia, n = 8, *p* = 0.019 two-way ANOVA, Fig. 2L, Additional file 1). Furtherly, we corroborated that overexpression of PSD95 reversed the decreased density of apical dendritic spines in the hippocampal region (significant difference between AAV-NC hypoxia vs. AAV-PSD95 hypoxia, *p* = 0.046 two-way ANOVA, Fig. 2M, N, Additional file 1). Therefore, increasing PSD95 rescues hypobaric hypoxia induced memory deficits and dendritic spine morphology injury.

### PSD95 is post-transcriptionally regulated by Cirbp under hypoxic condition

**Fig. 3:**
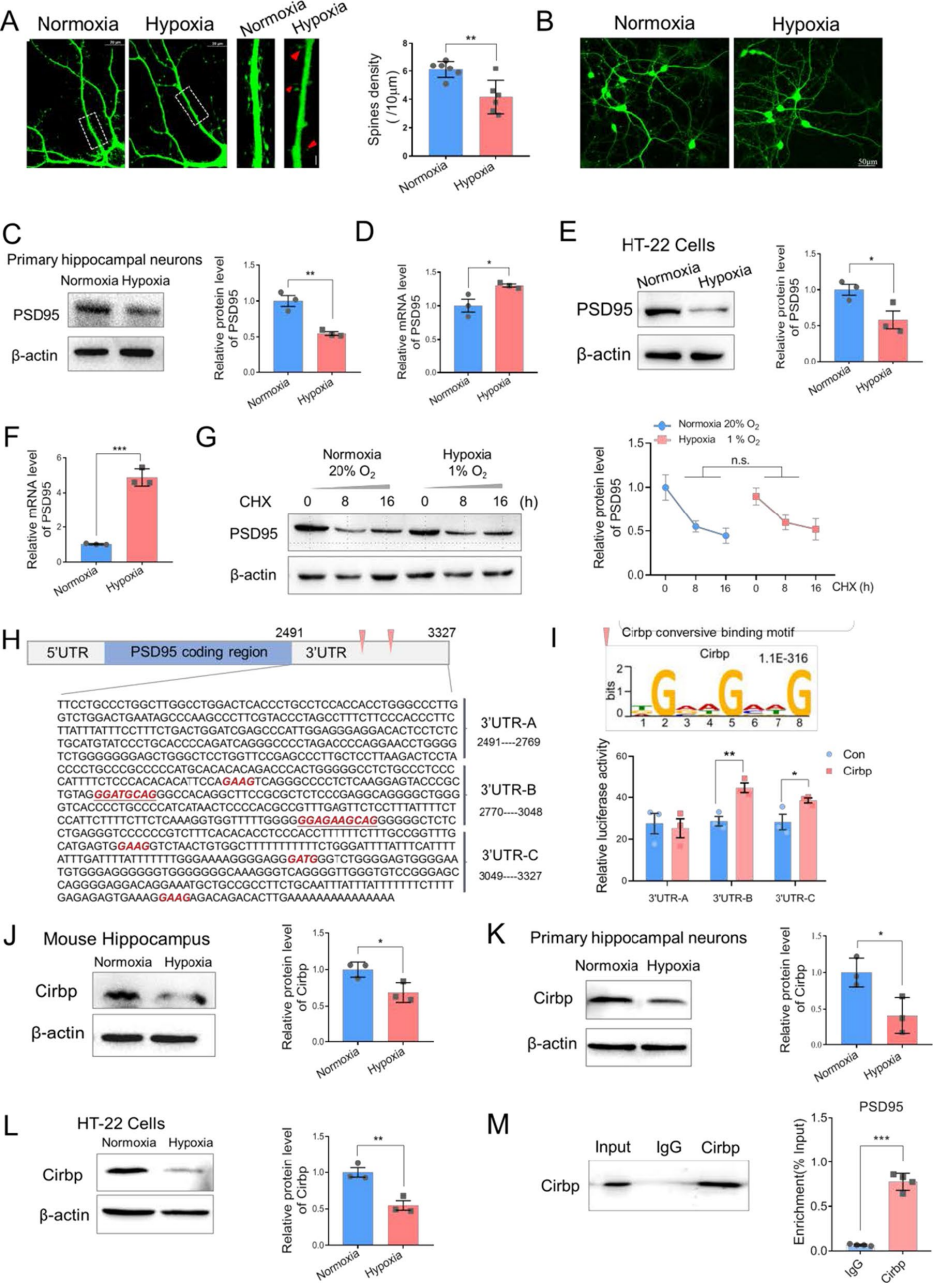
Hypoxic exposure resulted in aberrant dendritic spine morphology and PSD95 expression in vivo and Cirbp regulated PSD95 expression through 3′UTR binding. **A** Left: representative images of hippocampal neurons with hypoxia or normoxia exposure, scale bar = 10 μm. Middle: enlarged images of hippocampal neurons, red triangle indicated the elimination of spines or filopodia, scale bar = 5 μm. Right, the quantification of spines density (n = 6 per group, Student’s *t*-test, ± SEM). **B** Morphological changes of primary hippocampal neurons under hypoxic exposure, scale bar = 50 μm. **C** Representative immunoblotting (left) and quantitative analysis (right) of PSD95 in primary hippocampal neurons under hypoxic exposure (n = 3 biological replicates, Student’s *t*-test, ± SEM). **D** The relative mRNA levels of *PSD95* in primary hippocampal neurons under hypoxic exposure (n = 3 biological replicates, Student’s *t*-test, ± SEM). **E** Representative immunoblotting and quantitative analysis of PSD95 in HT-22 cells following normoxic or hypoxic exposure (n = 3 biological replicates, Student’s *t*-test, ± SEM). **F** The relative mRNA levels of *PSD95* in HT-22 cells (n = 3 biological replicates, Student’s *t*-test, ± SEM). **G** PSD95 expression after CHX treatment based on Western blot and quantitative analysis (n = 3 biological replicates, paired Student’s *t*-test, ± SEM). **H** RBP binding motif distributing in *PSD95* 3′UTR and construction of a series of luciferase reporter vectors containing *PSD95* 3′UTR fragments. **I** Luciferase activity assays to examine the functional RBP motif sites in the *PSD95* 3′UTR regulated by Cirbp (n = 3 biological replicates, Student’s *t*-test, ± SEM). **J**–**L** The protein levels of Cirbp in mice hippocampus (**J**), primary hippocampal neurons (K) and HT-22 cells (**L**) following hypoxia exposure (n = 3 biological replicates, Student’s *t*-test, ± SEM). **M** RNA immunoprecipitation (RIP) followed by RT-PCR in HT-22 cells (n = 4 biological replicates, Student’s *t*-test, ± SEM). *p < 0.05, **p < 0.01, ***p < 0.001

To elucidate the mechanism underlying the dendritic spine abnormalities caused by exposure to hypobaric hypoxia, we evaluated spine morphology in primary hippocampal neurons. Primary cultured neurons were transfected with fluorescent-GFP and cultured in the normoxia (21% O_2_) and hypoxia (1% O_2_) conditions for 24 h. Consistent with our experiments in vivo, we found that hypoxic exposure resulted in reduction in spine density in cultured neurons (6.13 ± 0.59 in Normoxia vs. 4.17 ± 1.18 for Hypoxia, *p* = 0.005 Student’s *t*-test, Fig. 3A, Additional file 1). There were no cell death or swelling in morphological observation (Fig. 3B). Level of PSD95 protein was concomitantly and significantly decreased in the hypoxic group when compared with the control group in primary hippocampal neurons (n = 3 biological replicates, *p* = 0.005 Student’s *t*-test, Fig. 3C, Additional file 1, 2) and HT-22 cells (n = 3 biological replicates, *p* = 0.045 Student’s *t*-test, Fig. 3E, Additional file 1, 2). However, under hypoxic conditions, inconsistent with the noted down-regulation of protein level, *PSD95* expression at the RNA level was elevated in primary hippocampal neurons and HT-22 cell lines (n = 3 biological replicates, *p* = 0.039 and *p* < 0.001 respectively, Student’s *t*-test, Fig. 3D, F, Additional file 1). These results indicated that under hypoxic conditions, the expression of PSD95 is not regulated at transcriptional level and may be determined by post-transcriptional level, which may lead to the compensatory increasing of *PSD95* mRNA. To evaluate whether PSD95 expression is controlled through protein degradation mechanism, we applied Cycloheximide (CHX) treatments (5 μg/ml, 0, 8, 16 h) under hypoxic exposure to determine the PSD95 protein degradation rate. Western blot results verified that PSD95 protein was dramatically down-regulated upon CHX treatment, and there were no statistically significant differences in its protein levels upon normoxic or hypoxic conditions (n = 3 biological replicates, paired Student’s *t*-test, Fig. 3G, Additional file 1, 2). It would be noted that the RNA level of *PSD95* decreased in murine hippocampus (Fig. 1B) and increased in primary neurons and HT-22 cells (Fig. 3D, F) after hypoxia exposure. It doesn’t seem that the RNA degradation mechanism contribute to the regulation of PSD95 expression in hypoxia condition. We speculated that hypoxia exposure suppressed PSD95 expression at translational level and different mechanisms modulated PSD95 mRNA stability in vivo and in vitro respectively, which requires further investigation.

As RNA-binding protein (RBP) plays a core role in post-transcriptional regulation, we then analyzed RBP binding motif distribution in *PSD95* mRNA, noting several Cirbp binding sites in the 3′UTR region of *PSD95* mRNA (Fig. 3H). To further verify that Cirbp regulates the PSD95 expression through binding to its motif in 3′UTR region, we divided the *PSD95* 3′UTR into three fragments and constructed a series of luciferase reporter vectors (3′UTR A, B and C fragments). Cirbp motif sites were only present in B, and C fragments, and the luciferase assay showed that Cirbp significantly increased the luciferase activity of fragment B and C, but did not affect the activity of fragment A (n = 3 biological replicates, Student’s *t*-test, Fig. 3I, Additional file 1). The protein Cirbp in murine hippocampus was down-regulated under hypoxic condition (n = 3 biological replicates, Student’s *t*-test, Fig. 3J, Additional file 1, 2). Similar findings were observed both in primary cultured hippocampal neurons (n = 3 biological replicates, Fig. 3K, Additional file 1, 2) and HT-22 cells (n = 3 biological replicates, Fig. 3L, Additional file 1, 2). Next, we performed RNA immunoprecipitation (RIP) to isolate mRNA bound to endogenous Cirbp in HT-22 cells and utilized specific primers to identify the Cirbp motif regions by RT-PCR analysis. The data demonstrated that the *PSD95* 3′UTR region was highly enriched in Cirbp immunoprecipitated samples compared to IgG controls (n = 4 biological replicates, *p* < 0.001 Student’s *t*-test, Fig. 3M, Additional file 1). Mechanistically, these finding suggest that Cirbp binds to *PSD95*-3′UTR, and post-transcriptionally regulates its expression.

### Cirbp up-regulates PSD95 expression and improves hypoxia induced dendritic spines impairment

**Fig. 4:**
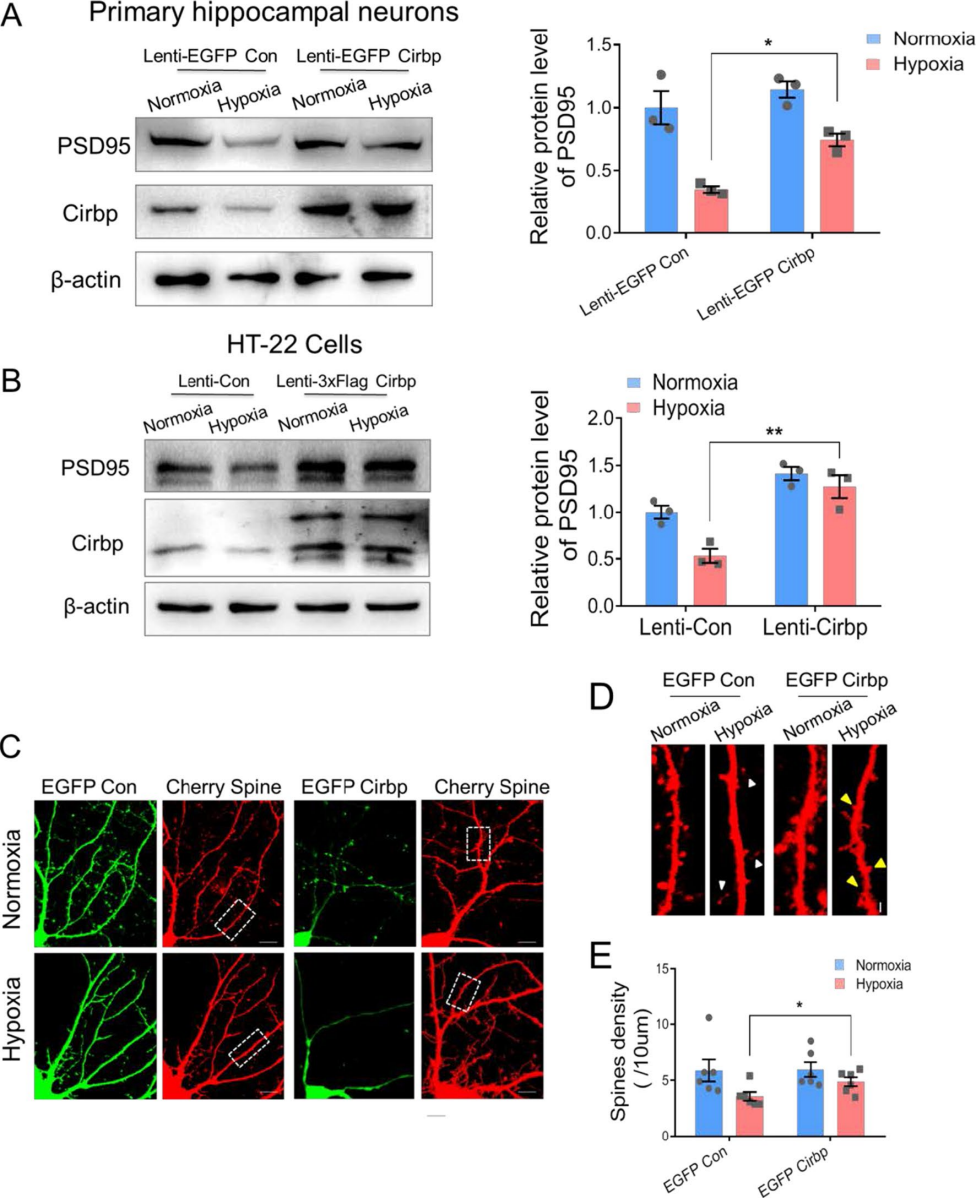
Effects of over-expression of Cirbp in primary hippocampal neurons exposed to hypoxia on PSD95 expression and the spine morphology. **A**, **B** The protein levels of Cirbp and PSD95 in Lenti-EGFP Con and Lenti-EGFP Cirbp infected primary hippocampal neurons (**A**) and HT-22 cells (**B**) (n = 3 biological replicates, two-way ANOVA, ± SEM). **C** Fluorescence images of EGFP Cirbp and mCherry-3FLAG lentivirus infected primary hippocampal neurons under normoxia or hypoxia exposure, scale bar = 10 μm. **D** The zoomed picture of dendritic spines, corresponds to the in the white frame of via laser confocal microscopy (white triangle indicated filopodia and yellow triangle indicated the site of spine formation, scale bar = 5 μm). **E** The statistical analysis of dendritic spine density (6 neurons/3 mice per group, two-way ANOVA, ± SEM). *p < 0.05

To examine whether Cirbp influences the expression of PSD95 to regulate hypoxia induced structural change of dendritic spines, we showed that the protein level of PSD95 was rescued by overexpression of Cirbp following hypoxic exposure in hippocampal neurons and HT-22 cells (significant difference between Lenti-EGFP Con hypoxia and Lenti-EGFP Cirbp hypoxia, n = 3 biological replicates, two-way ANOVA, Fig. 4A, B, Additional file 1, 2). Western blot analysis in pEGFP-Cirbp lentivirus infected primary hippocampal neurons showed that overexpressed Cirbp significantly reversed the hypoxia induced reduction in spine density (significant difference of spines density between Lenti-EGFP Con hypoxia and Lenti-EGFP Cirbp hypoxia, two-way ANOVA, Fig. 4C–E, Additional file 1). These results presented that Cirbp modulates PSD95 expression under hypoxia and is a novel key factor in protecting hypoxia-induced dendritic spine morphology aberrations.

### Overexpressed Cirbp protects hypoxia induced memory capacity dysfunction

**Fig. 5:**
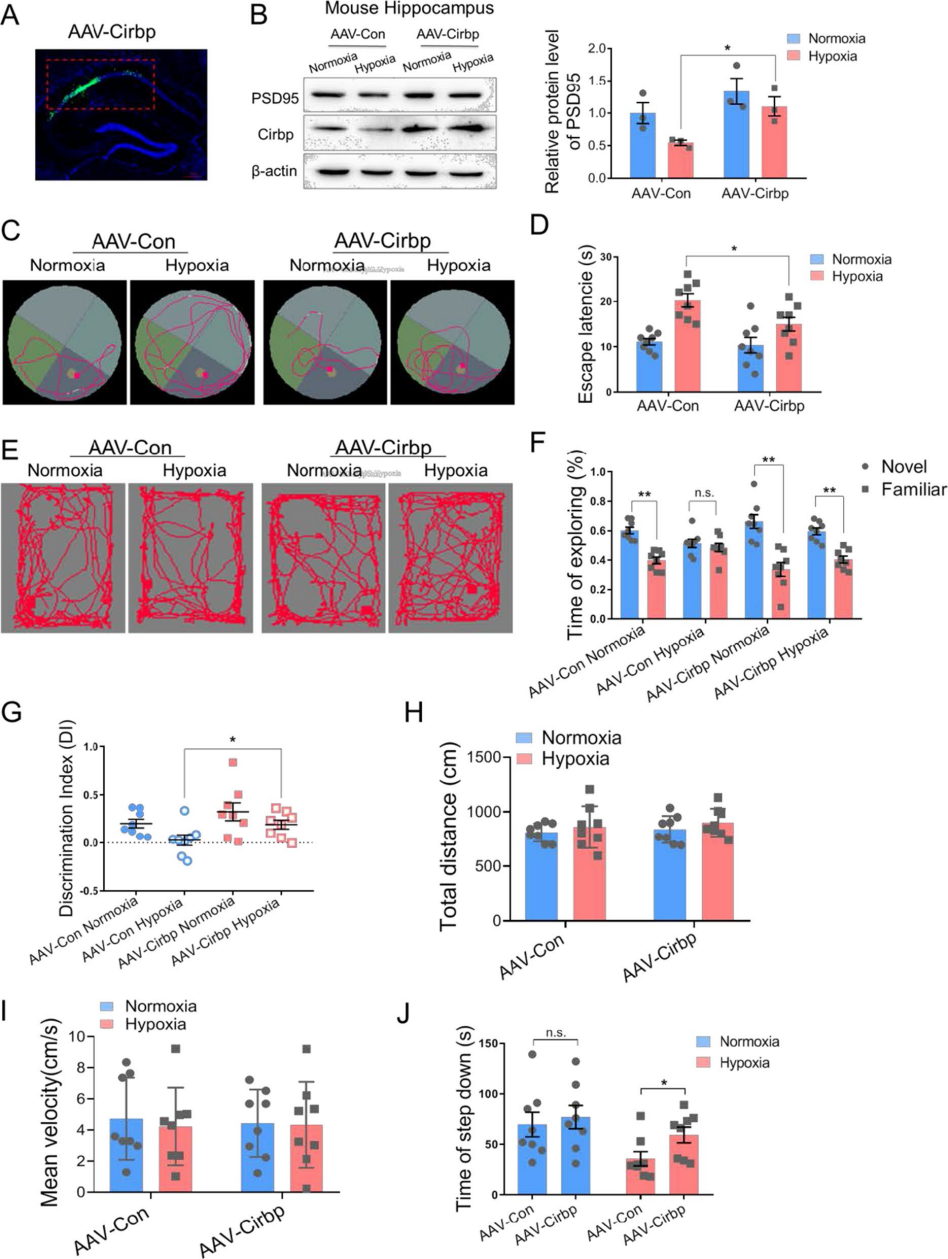
Ectopic expression of Cirbp alleviated memory dysfunction in hypobaric hypoxia exposed mice. **A** Fluorescence image of mouse hippocampal CA1 region (red frame area) after stereotactic injection showing Cirbp expression (green) and Nucleus (blue), scale bar = 50 μm. **B** Protein expression of PSD95 in mouse hippocampus following hypoxia exposure after over-expressing Cirbp (n = 3 biological replicates, two-way ANOVA, ± SEM). **C**, **D** Representative tracking plots (**C**) and the escape latency (**D**) of mice under indicated treatment in MWM test (n = 8, two-way ANOVA, ± SEM). **E**–**I** Representative locomotion tracking plots (**E**), exploring time on new objects (**F**), discriminate index (**G**), total distance (**H**) and mean velocity (**I**) of mice under indicated treatment in NORT test (n = 8, two-way ANOVA, ± SEM). **J** The latency time of step down in SIAT test (n = 8, two-way ANOVA, ± SEM). *n.s.* no significant, *p < 0.05, **p < 0.01

It is interested whether Cirbp expression could rescue the cognitive capacity defect in hypoxia exposed mice. AAV-Cirbp was injected into the murine hippocampal region to establish the Cirbp expressing in vivo model. Fluorescence imaging showed that the spontaneous GFP-fluorescence was localized in the hippocampal CA1 region (Fig. 5A). The blot results showed that Cirbp expression was increased in the hippocampus, and that hypoxia-induced PSD95 downregulation was reversed upon hypoxic exposure (significant difference between AAV-Con hypoxia and AAV-Cirbp hypoxia, n = 3 biological replicates, *p* = 0.017 two-way ANOVA, Fig. 5B, Additional file 1, 2). Furthermore, MWM and SIAT results revealed that overexpressing Cirbp in the hippocampus recovered the hypoxia-induced memory impairment, with decreased escape latency (significant difference between AAV-Con hypoxia and AAV-Cirbp hypoxia, n = 8, *p* = 0.040 two-way ANOVA, Fig. 5C, D, Additional file 1), and increased step-down time (significant difference between AAV-Con hypoxia and AAV-Cirbp hypoxia, n = 8, *p* = 0.015 two-way ANOVA, Fig. 5J, Additional file 1). The NORT task showed that there was no significant difference in total distance and the mean speed among mice in these groups (Fig. 5H, I, Additional file 1). However, the exploring time for new objects (Fig. 5F, Additional file 1) and the discrimination index (DI) (significant difference between AAV-Con hypoxia and AAV-Cirbp hypoxia, n = 8, *p* = 0.034 two-way ANOVA, Fig. 5G, Additional file 1) in AAV-Cirbp Hypoxia group were significantly greater than in the AAV-Con Hypoxia group. These results suggested that overexpression of Cirbp retrieved memory damage caused by hypoxic exposure in mice.

**Fig. 6:**
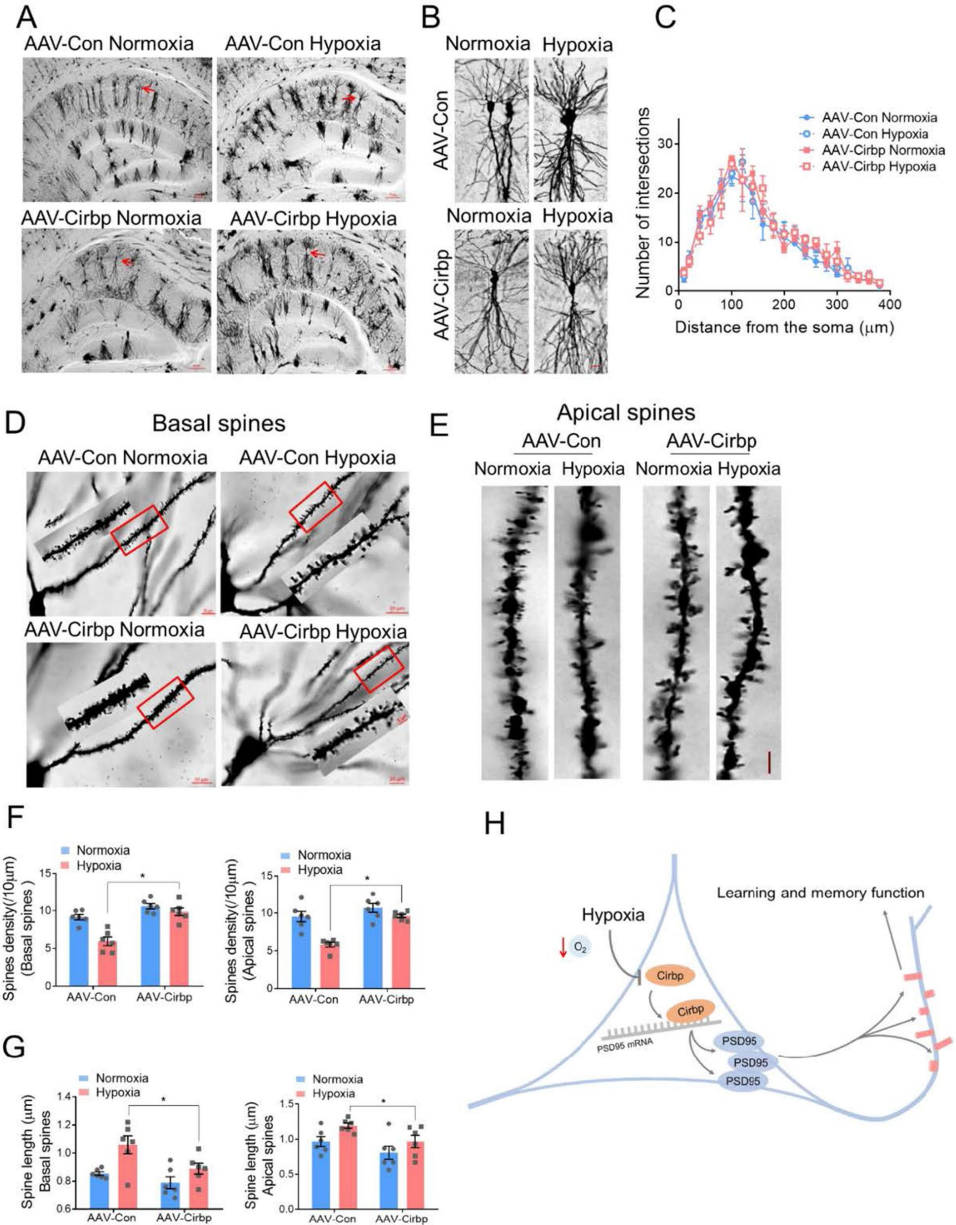
Cirbp alleviated hypoxia-induced dendritic spine abnormalities in mice hippocampus. **A**, **B** Golgi staining of mice hippocampus (A, scale bar = 50 μm) and of CA1 pyramidal neurons (B, scale bar = 20 μm). **C** Number of intersections of pyramidal neuron reconstruction (three mice per group, two-way ANOVA, ± SEM). **D** Representative Golgi staining morphology of apical dendritic spines in CA1, scale bar = 20 μm (red frame indicated target dendritic, scale bar = 5 μm). **E** Representative morphological images of apical dendritic spines in CA1, scale bar = 5 μm. **F** Quantitative analysis of basal and apical spines density (6 neurons/3 mice per group, two-way ANOVA, ± SEM). **G** Quantitative analysis of basal and apical neck length (6 neurons/3 mice per group, two-way ANOVA, ± SEM). **H** A model for key role of Cirbp-PSD95 axis in hypoxia-induced hippocampal neuron dendritic spine abnormality. *p < 0.05

Moreover, Golgi staining showed that there was no significant change in dendritic branches in hippocampal CA1 region in each of the groups (Fig. 6A–C, Additional file 1). Under hypoxic condition, Cirbp overexpression significantly reversed the density reduction in basal dendrites and apical hippocampal dendrites (*p* = 0.0124 and *p* = 0.0138 respectively, two-way ANOVA, Fig. 6D–F, Additional file 1), and decreased the length of spines’ necks under hypoxia (*p* = 0.0145 and *p* = 0.0201 respectively, two-way ANOVA, Fig. 6G, Additional file 1). These findings corroborated that overexpression of Cirbp attenuated the hypoxia-induced dendritic spine plasticity disorder.

Taken together, our results demonstrate that Cirbp participate in hypoxia induced memory disability and spines morphology aberration through post-transcriptionally modulating PSD95 expression in murine hippocampus (Fig. 6H).

## Discussion

The effect of chronic hypobaric hypoxia on the central nervous system is multifaceted, including impaired cerebral auto-regulation, gliovascular deficit, oxidative stress, and neuroinflammation, ultimately converting into cognitive deficits [17]. Abnormal structure of dendritic spines is a common phenomenon in central nervous system disorders, which are often associated with disease neuropathology [18, 19], but their role in the etiology of altered cognitive function under hypoxia is not fully understood. In this study, we examined hypobaric hypoxic exposure on cognitive capacity, dendritic arborization of neuron and spines number in hippocampal region, which plays an important role in processing and storing learned information [6]. It was observed that mice exposed to hypobaric hypoxia exhibited hippocampus mediated memory deficits with accompanying abnormal neuronal spines morphology. Furthermore, the level of synaptic protein PSD95 was suppressed by hypoxic exposure, and increasing PSD95 mitigated the deleterious effects of hypoxia on memory dysfunction and dendritic spine abnormities. Biochemical and molecular analysis indicated that the level of PSD95 was regulated post-transcriptionally. By analyzing RNA binding protein motif, we found that there are several Cirbp binding sites in 3′UTR region of *PSD95* mRNA and Cirbp regulates PSD95’s expression via binding with its 3′UTR. Finally, abnormal cognitive performance and dendritic spine density under hypoxia were partially restored after expression of Cirbp. Collectively, these results delineate a Cirbp-PSD95 axis in the hippocampus that rivals dendritic spine remodeling and cognitive impairment in hypoxia exposed mice (Fig. 6H).

As the main environmental characteristics of the plateau, hypobaric hypoxia has attracted more attention to the effects and mechanisms of human health. In addition to the respiratory and circulatory system, brain is also a sensitive target organ for hypoxia-induced injuries. Long-term hypoxia stress leads to attention deficit, memory loss, and cognitive dysfunction, even increased risk of neurodegenerative diseases, such as Alzheimer's disease (AD) [20–22]. The hippocampus is primarily responsible for learning-memory process and is closely related to short-term memory formation, which is the most sensitive to hypoxia induced damage [2, 23, 24]. Dendritic spines are small protrusion emerging from the dendritic shaft in many neurons, and represent the postsynaptic element of most excitatory glutamatergic synapses [6]. These are highly dynamic structures, and changes in the number, size and shape of spines are correlated with modifications in synaptic strength [25, 26]. An exuberant excess of spines having an immature, long and thin morphology is a phenotype related with mental disability [27], and stable dendritic spines can enhance memory storage capacity by establishing new synaptic connections [28]. The formation or enlargement of dendritic spines affects the enhancement of synaptic connections, while the shrinking or disappearance of dendritic spines represents a weakening of synaptic connections [29]. Our studies showed that hypoxia-exposed animals had deficits in memory extraction and presentation, consistent with impaired learning and memory ability in human. Further results indicated that normal spine remodeling process could be disrupted by the hypoxic exposure, with less stable and more immature dendritic spines in mice. Studies on primary hippocampal neurons showed that hypoxia caused dentritic spines density decreasing. These observations of hypoxia-inhibited development of dendritic spines were consistent with previous studies [30, 31], which demonstrate that disturbance of spine morphology after hypobaric hypoxia might be associated with impairment of cognitive functions.

RBPs have been identified as key molecules in many diseases including neurodegenerative disorders [32]. Cirbp belongs to a family of cold shock proteins and has been shown to play a positive role in hypothermia-induced neuroprotection [33], sleep homeostasis [34], circadian [35], cardioprotection [10], inflammatory responses of shock and sepsis [36] and neonatal brain injury [37]. Most of the researches on Cirbp focused on its role upon cold exposure. In this study, we found that expressing Cirbp in murine hippocampus alleviated the damaged memory capacity and spine structure caused by hypoxia, which specified the neuroprotective function of Cirbp. A previous study confirmed a protective role for Cirbp in hypoxia-induced nerve injury [11], consistent with our study. The cognitive neurological manifestation of Cirbp knockout animal is an intriguing problem. In a recent work, Jacob et al., exposed Cirbp^−/−^ mice and wild type mice to alcohol exposure and assessed behavioral changes through object place memory test, which indicates the spatial memory ability of animals [38]. The results showed that WT and Cirbp^−/−^ mice had similarly positive discrimination ratios and alcohol treated WT mice showed significantly impaired spatial memory, whereas Cirbp^−/−^ mice were not impaired in OPM task. This study indicated that Cirbp gene knockout has a neuroprotective effect when compared to WT mice, which is inconsistent with our study. One of possible explanations is the difference between alcohol neurotoxicity and hypoxic neural injury. The behavior of Cirbp^−/−^ mice under hypoxic condition needs further investigation and this part of research work is being carried out by us. Our findings are the first to demonstrate that Cirbp post-transcriptionally regulates PSD95 expression to protect hypoxia induced spine remodeling defect. These findings shed light on the important post-transcriptional regulatory role of RNA-binding proteins in controlling dendritic spine homeostasis and learning-memory ability.

PSD95, as the main elements of chemical synapses, interacts with glutamate receptors, cell adhesion molecules and cytoskeleton elements. As it is well known, PSD95 can modulate the stability of basal synapses and the activity-dependent structural plasticity of PSD and excitatory chemical synapses caused thereby [39]. Previous studies have shown that PSD95 was post-transcriptionally regulated at excitatory synapses and there are several neurological disorders, including AD, in which impairments in the normal function of PSD95 are associated with post-translational modifications [40]. PSD95 is also a target of several signaling pathways that induce post-translational modifications, including palmitoylation, phosphorylation, ubiquitination, nitrosylation, and neddylation. These modifications determine the synaptic stability and function of PSD95, and thus regulate the fates of individual dendritic spines in the nervous system [41]. Moreover, PSD95 has been shown to facilitate post-translational modifications as to modulate its postsynaptic localization within the dendritic spine, thereby influencing chemical synaptic transmission regulation in the CNS [41]. The expression of PSD95 is controlled and restricted by at least two mechanisms: the action of miR-125a and the degradation of PSD95 mRNA, the latter of which is mediated by two polypyrimidine tract binding (PTB) proteins. The overall result is inhibition of excitatory synapse formation as well as neuronal maturation [42]. The synaptic localization of PSD95 can be regulated by various post-translational modifications, depending on developmental stage, synaptic activity, and disease [41]. Here we found that PSD95 was significantly reduced at its protein level without the change of its RNA level and there was no difference in PSD95 degradation rates under hypoxic and normoxic conditions, which indicated that the RNA stability mechanism and protein degradation machines is not involved in PSD95 expression influenced by hypoxia. Thus, these findings suggested that RNA binding protein Cirbp binds with *PSD95* mRNA 3′-UTR and plays a crucial role in its post-transcriptional regulation under hypoxic situation.

Combined, the present data offer novel understanding of regulatory molecules of cognitive dysfunction under long-term exposure to high altitude environment. Our data demonstrated that Cirbp alleviates hypoxia-induced memory impairment and binds *PSD95* mRNA 3′-UTR, forming a novel signaling pathway to balance dendritic spine remodeling. Thus, Cirbp-PSD95 axis acts as a key protective regulator in hypoxic stress, offering a potential new therapeutic site in mitigating the aberrant effects of hypobaric hypoxia.

## Materials and methods

### Animals and hypoxic exposure

All procedures involving animals were in accordance with the procedures outlined in the “National Institutes of Health guide for the care and use of Laboratory animals, Eighth Edition” (https://grants.nih.gov/grants/olaw/Guide-for-the-Care-and-use-of-laboratory-animals.pdf), and were approved by the Institutional Animal Care and Use Committee of the Fourth Military Medical University (01 May 2017, 05 Feb 2021). 8-week C57BL/6 mice were obtained from the animal center of the Fourth Military Medical University and were kept under constant temperature (22–24 °C) and 12 h light–dark cycle with free access to food and water. Mice were randomly assigned into two groups (normoxia and hypoxia). After five days of adaptation, the normoxic (control) group was kept at normal atmospheric pressure, and the hypoxic group was placed in were placed in the decompression chamber (Fenglei Co. Ltd., China) for continuous hypobaric hypoxic exposure equal to an altitude of 6000 m (barometric pressure = 349 mmHg and partial O_2_ pressure = 8–9%) for 14 days as described in our previous report [43].

### Cell culture and hypoxia exposure

Primary hippocampal neurons were cultured as previously described [44]. Embryonic day 18 C57BL/6 mice embryos were placed in pre-cooled HBSS Hanks (Gibco, USA) and stripped off the meninges carefully under stereo-microscope (Nikon, Japan). Hippocampi were isolated by removing the bleeding spots, and treated with trypsin (12604013, Gibco, USA). Hippocampal dissociated cells were cultured in Neurobasal medium (Gibco, USA) with B27 (17504044, Gibco, USA), L-Glutamine (G6392-1VL, Sigma-Aldrich, USA), streptomycin and penicillin (MP Biomedicals, USA), and half-liquid exchange was performed every 3 days. For assessment of dentric spines, neurons were infected with p-EGFP lentivirus (Obio, China). Mouse hippocampal neuronal HT-22 cells were cultured as detailed in our previous study [45].

Cells were exposed to hypoxia in a humidified microaerophilic incubation system (DWS-H85, DonWhitley, UK) filled with 1% O_2_ and 5% CO_2_ as described in previous studies [11, 43]. Culture medium was placed in the hypoxia incubator for more than 9 h in advance.

### Morris water maze (MWM) test

Animals were trained in the hidden platform version of the MWM task as described in our publications [46].The hidden platform training was performed for 5 days straight (4 trials per mouse, per day). Each animal was placed in one of four chosen starting locations in the pool and had 60 s to locate the hidden platform. If an animal failed to find the platform, it was placed on top of it and had to remain there for 20 s before being put back into its cage by the experimenter. After 5 days of training, one probe trial was performed per mice and each mouse searched for 60 s. All trials were recorded via a digital camera connected to Enthovision ET software and DigiBehave system (Xinruan, China) which was used to record the animals’ behaviors.

### Novel object recognition test (NORT)

The novel object recognition protocol was developed based on previous procedures [47]. After 30 min room acclimation, mice were placed individually in open field enclosure and allowed to explore the empty environment. Then mice were placed in the open field enclosures (50 mm × 35 mm × 20 mm) in which two identical objects (A1 and A2) were placed equidistant from the corners of the enclosure, allowed 3 min to explore freely. On the second day, one of the A objects was replaced with B object before 3 min exploration. The familial object exploration time (T_A_) and novel object exploration time (T_B_) were recorded on the video tracking system (DigiBehave system, Xinruan) and discriminate index (DI) was calculated [DI = (T_B_ − T_A_)/(T_B_ + T_A_)]. The area was wiped clean with 70% ethanol between each test to minimize residual odor cues effect.

### Step-down inhibitory avoidance test (SIAT)

The step down inhibitory avoidance test was performed following previous protocol [48]. During the training session, the animals were placed on a circular platform and received a 2 s, 0.4 mA scrambled foot shock right after stepping down onto the grid with all 4 paws. Mice were trained for three times and tested after 2 h interval. If the animal did not step down the platform after 150 s, the final time was recorded as 150 s.

### Golgi staining and morphological analysis

For Golgi staining as our previous description [49], mice were deeply anesthetized with sodium pentobarbital (100 mg/kg, i.p.) and transcardially perfused with 0.9% saline. Whole brains were processed using an FD Rapid GolgiStain™ Kit (A:B = 1:1) (FD Neuro Tech-Inc, USA) and sliced at 100 μm using a vibratome (VT1000S, Leica). Sections were collected on gelatinized glass slides and immersed in Golgi solution in the dark at room temperature for 15 min. The tissue sections were dehydrated with 50%, 75%, 95%, and 100% ethanol for 4 min each. The slides were cleared with xylene (Fuyu, China) at room temperature in the dark, and coverslip with DPX Mountant (Sigma-Aldrich, USA). An optical microscope (Zeiss, Germany) was used to image the slides, with low magnification (× 20) for dendritic tree and high magnification (× 100) for spine reconstruction. Quantitative differences in the density of dendritic spines on individual dendritic branch orders between conditions was determined as previously described [49].

### RNA extraction and real-time quantitative PCR

Total RNA were extracted with Trizol reagent (Invitrogen, USA), and cDNAs were synthesized with the PrimeScript™ RT Master Mix (RR036A, TAKARA, Japan). Real-time quantitative PCR was performed using TB Green® Premix Ex Taq™ II assays (820A, TAKARA, Japan) in FAST-7500 system (Applied Biosystems, USA) following standard protocols [46]. The *Mus musculus PSD95* cDNA fragment were amplified by primers 5′-TGC ATC TCT GCG AAG CAA CC-3′ and 5′-GCG TCA ATT ACA TGA AGC ACA TCC-3′), and two oligonucleotides 5′-AGC CAT GTA CGT AGC CAT CC-3′and 5′-GCT GTG GTG GTG AAG CTG TA-3′ were used as the specific primers to amplify *Mus musculus β-actin* cDNA as a loading control. The 2^ΔΔCt^ values were calculated [50] and data were normalized to *β-actin*.

### Plasmid construction and transfection

Cirbp plasmid (pLenti-EF1a-EGFP-P2A-Puro-CMV-Cirbp-3Flag) and pEGFP-N2 control vector were constructed by Obio Technology Company (Shanghai, China). Transfection complexes, consisting of 5 μg plasmid DNA and 10 μl Lipofectamine reagent (Thermo Fisher Scientific) were added to the 6 cm wells in Opti-MEM® Medium (Invitrogen, USA). Cells were replaced with the DMEM-High Glucose (Gibco, USA) after 6 h and the follow-up experiments continued.

### In vitro lentivirus infection

Primary hippocampal neurons were seeded in cell dishes covered by polylysine at a density of 2 × 10^5^, cultured for three days for semi-liquid exchange, and directly added to the virus solution with pLenti-CMV-EGPF-3FLAG (Obio Technology, China) or pLenti-CMV-mCherry-3FLAG (Obio Technology, China) and pHBLV-CMV-MCS-EF1-Zsgreen1-T2A-puro (Hanbio Biotechnology, China) on the fifth day according to the dose of MOI = 10. (Virus amount = cell number × MOI value/original virus titer) × 10^3^. The following day, the cell liquid was exchanged with fresh solution, and then continued to culture and keep changing every 3 days.

### Protein extraction and Western blot analysis

Western blot was conducted as previously described [51]. The following antibodies were used for the specific detection of individual protein signals: Cirbp, diluted 1:500, Proteintech10209-2-AP (UK); PSD95, 1:1000, Abcam18258, (UK); β-actin, 1:1000, Sigma A5441, (UK). The protein bands were visualized by ELC plus chemiluminescence (Qicai, China) and its intensities were determined by Image J software (National Institutes of Health, MD). Each protein band was normalized to β-actin values and expressed as the relative intensity ratio.

### Stereotactic injection of virus

The anesthetized C57BL/6 mice were mounted into the stereotaxic frame (RWD Life science, China). Needle was vertically and bilaterally lowered into the hippocampus (ML: ± 1.5; AP: − 2.18; DV: − 1.6 mm from bregma and skull). (0.3 μl (1 × 10^12^ VG/ml) of adeno-associated virus (AAV-CIRBP: pAOV-SYN-EGFP-P2A-CIRBP; AAV-PSD95: AAV-SYN-EGFP-P2A-Dlg-3FLAG) (Obio, Shanghai, China) was then bilaterally infused for 3 min and another 5 min allowed for diffusion before the injector was raised [52]. At the completion of viral infection, the incised skin covering the skull was grabbed with surgical staplers and animals were placed in a constant temperature heater at 32 °C to rewarm. Next, rats were returned to their home cages for 1-week recovery period. AAV viruses were expressed and detected after 3 weeks.

### Tissue fluorescence staining

At the end of the experiments, the animals were deeply anesthetized and transcardially perfused with 4% cold paraformaldehyde in 0.1 M phosphate buffer (PB). Brains were dissected out and post-fixed in the 30% sucrose solution for 3 days. Mice brains were sliced from the coronal part of the standard hippocampus (thickness 20 μm). 0.1 M PBS and 0.3% Triton-X100 (Kehao, China) was used for diluting all immunoreagents. Then, they were blocked with 3% BSA (MP Biomedicals, USA) for 30 min and dripped in primary antibody (NeuN, Abcam104224, UK) overnight at 4 °C, subsequently incubated with secondary antibodies conjugated with a fluorescent dye for 2 h. After washing, these sections were incubated with 4′,6-diamidino-2-phenylindole (DAPI, Beyotime, China) for 30 min at room temperature, and then covered with glycerin. Immunofluorescent images were captured on a fluorescence microscope (Zeiss, Germany).

### Luciferase reporter activity experiment

Three fragments of mouse *PSD95* (*dlg4*) mRNA 3′UTR [3′UTR-A(2491-2769nt), 3′UTR-B(2770-3048nt) and 3′UTR-C(3049-3327nt)] were cloned into pMIR-Report luciferase plasmid (GeneChem, China). For luciferase reporter assays, HT-22 cells were co-transfected with pMIR-REPORT luciferase plasmid with *PSD95* 3′UTR-A, -B and -C (500 ng/well), Cirbp plasmids (500 ng/well) or NC (control) plasmids (500 ng/well), and Prl-TK plasmid (50 ng/well) using Lipofectamine 2000 (Invitrogen, USA). The luciferase activity was determined with the Dual-Luciferase Reporter Assay System according to the manufacturer’s instructions as described [53].

### RNA binding protein immunoprecipitation (RIP)

Cell samples collected were lysed with an equal volume of complete RIP lysate buffer (100 μl RIP lysis buffer, 0.5 μl protease inhibitor mixture, 0.25 μl RNA inhibitor/unit). Cirbp antibody (5 μg) or IgG antibody (control) mixed with protein A/G Sepharose (Santa Cruz Biotechnology, Dallas TX) and continuously cultured at room temperature for 30 min. The cell lysate was added to a RIP immunoprecipitation buffer (RIP wash buffer, 0.5 M EDTA, ribonuclease inhibitor) containing a magnetic bead-antibody complex to obtain a mixed solution, which incubated at 4 °C for 9 h. In addition, an aliquot (10%) of supernatant were signed as Input and another for verification of Western-blot. Then, the immunoprecipitate were resuspended in proteinase K buffer (RIP wash buffer, 10% SDS, proteinase K) and incubated at 55° C for 30 min. RNA was isolated using Trizol reagent and subjected to RT-qPCR, using specific primers to detect the Cirbp binding.

### Data presentation and statistical analysis

All experimental data was analyzed using SPSS 20.0 software (IBM Corp, USA). The data were expressed as mean ± standard error of the mean (SEM). Differences between two groups were analyzed with Student’s *t*-test and data from the experiments involving several treatments were analyzed by multi-way ANOVA. A Tukey’s post hoc test revealed which groups differed significantly from one other. Probability values less than 0.05 were considered statistically significant.

## Supplementary Information

**Additional file 1.** Raw data used for quantified analyses of Figure 1–6.

**Additional file 2.** Entire western blot images of Figure 1–6..

## Data Availability

The datasets used and/or analyzed during the current study are available from the corresponding author on reasonable request.

## Abbreviations

AD: Alzheimer’s disease
ATP: Adenosine triphosphate
CHX: Cycloheximide
Cirbp: Cold-inducible RNA-binding protein
CNS: Central nervous system
DAPI: 4′,6-Diamidino-2-phenylindole
HA: High altitude
HH: Hypobaric hypoxia
LTP: Long-term potentiation
MWM: Morris water maze
NORT: Novel object recognition test
NR2A: *N*-methyl-d-aspartate 2A receptors
NR2B: *N*-methyl-d-aspartate 2B receptors
PSD: Post-synaptic density
RBP: RNA-binding protein
SIAT: Step-down inhibitory avoidance test
