# Supplementary material for: Cirbp-PSD95 axis protects against hypobaric hypoxia-induced aberrant morphology of hippocampal dendritic spines and cognitive deficits
Source: Mol Brain. 2021 Aug 21;14:129. doi: 10.1186/s13041-021-00827-1 (PMC8379783; doi:10.1186/s13041-021-00827-1)
Supplement: Supplementary file 2 — Additional file 2. Entire western blot images of Figure 1–6. [file 13041_2021_827_MOESM2_ESM.pdf]

Supplemental materials

Figure 2A. Mouse hippocampus

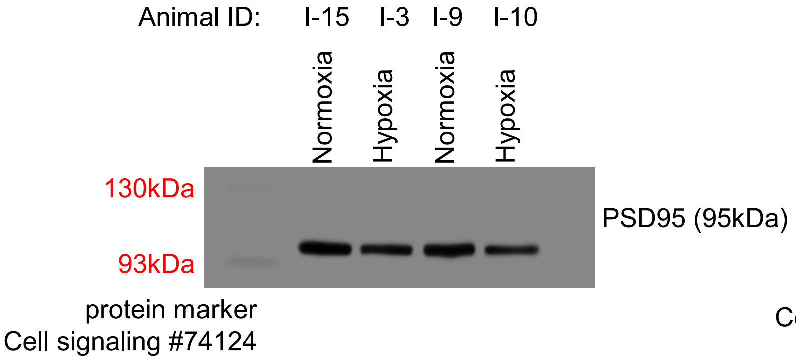

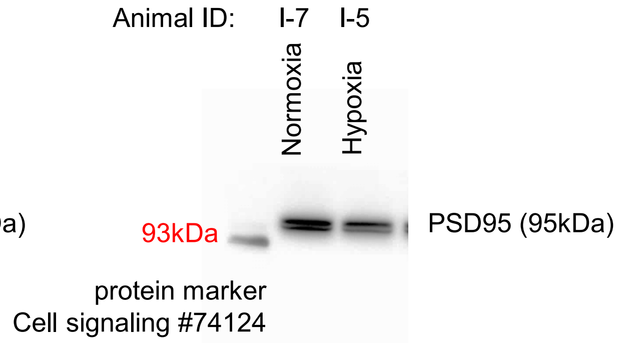

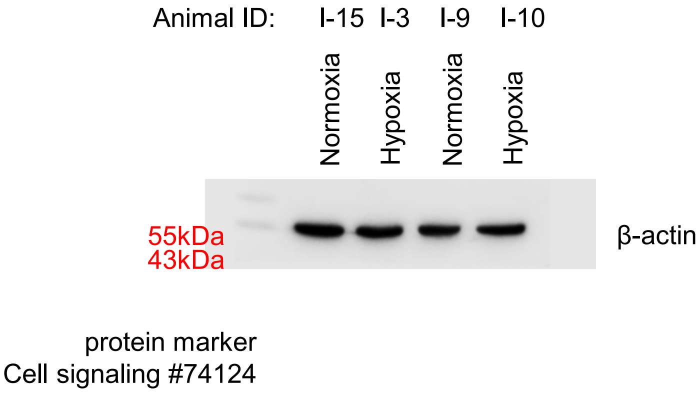

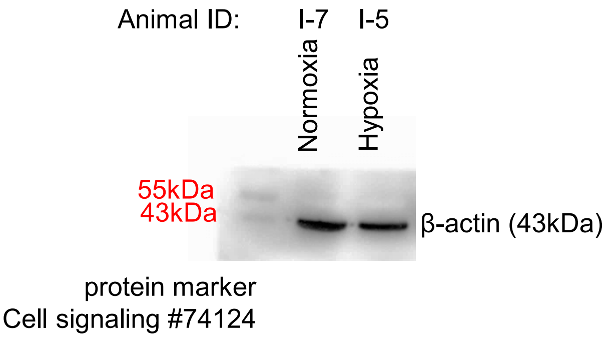

Figure 2O. Mouse hippocampus

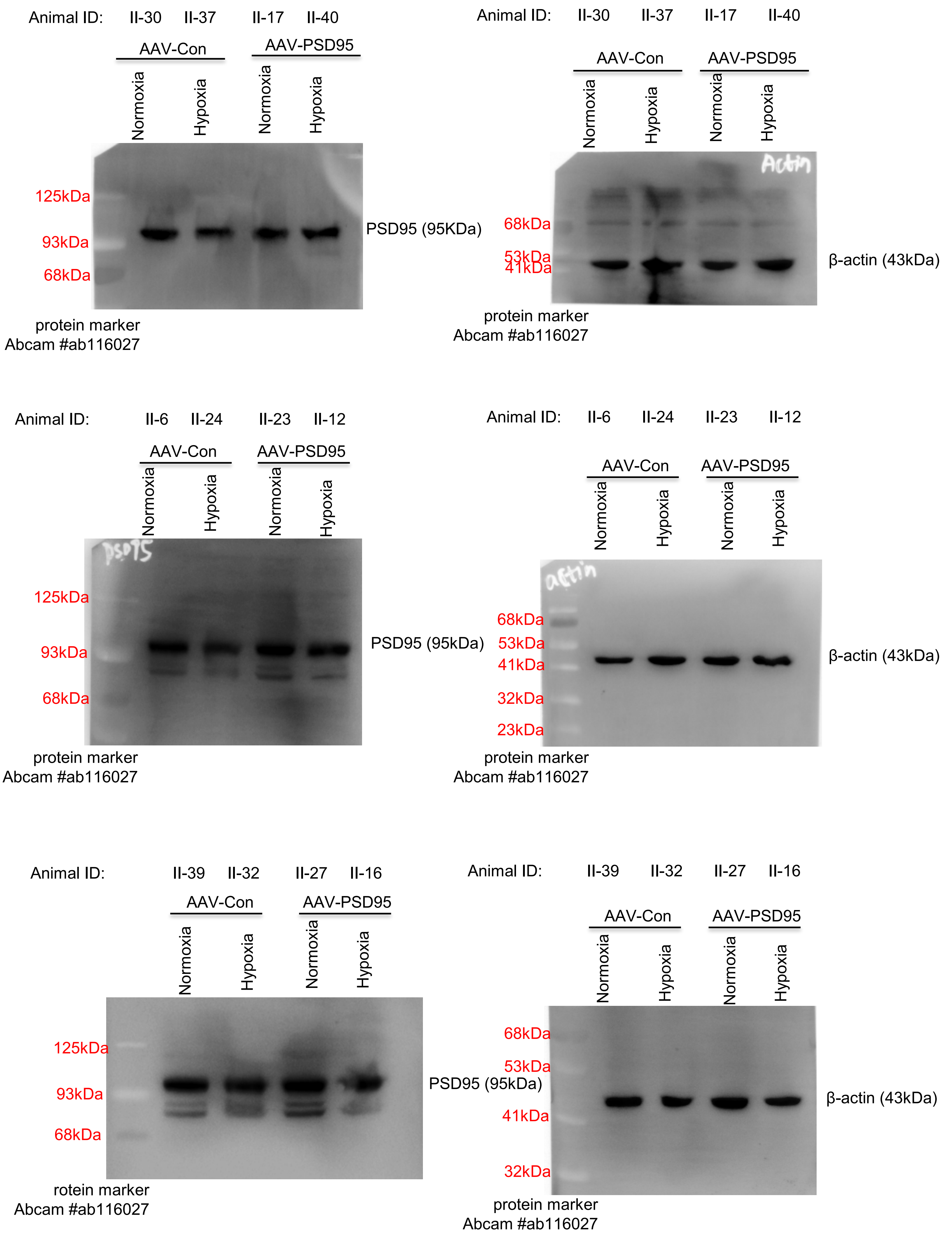

Figure 3C. Primary hippocampal neurons

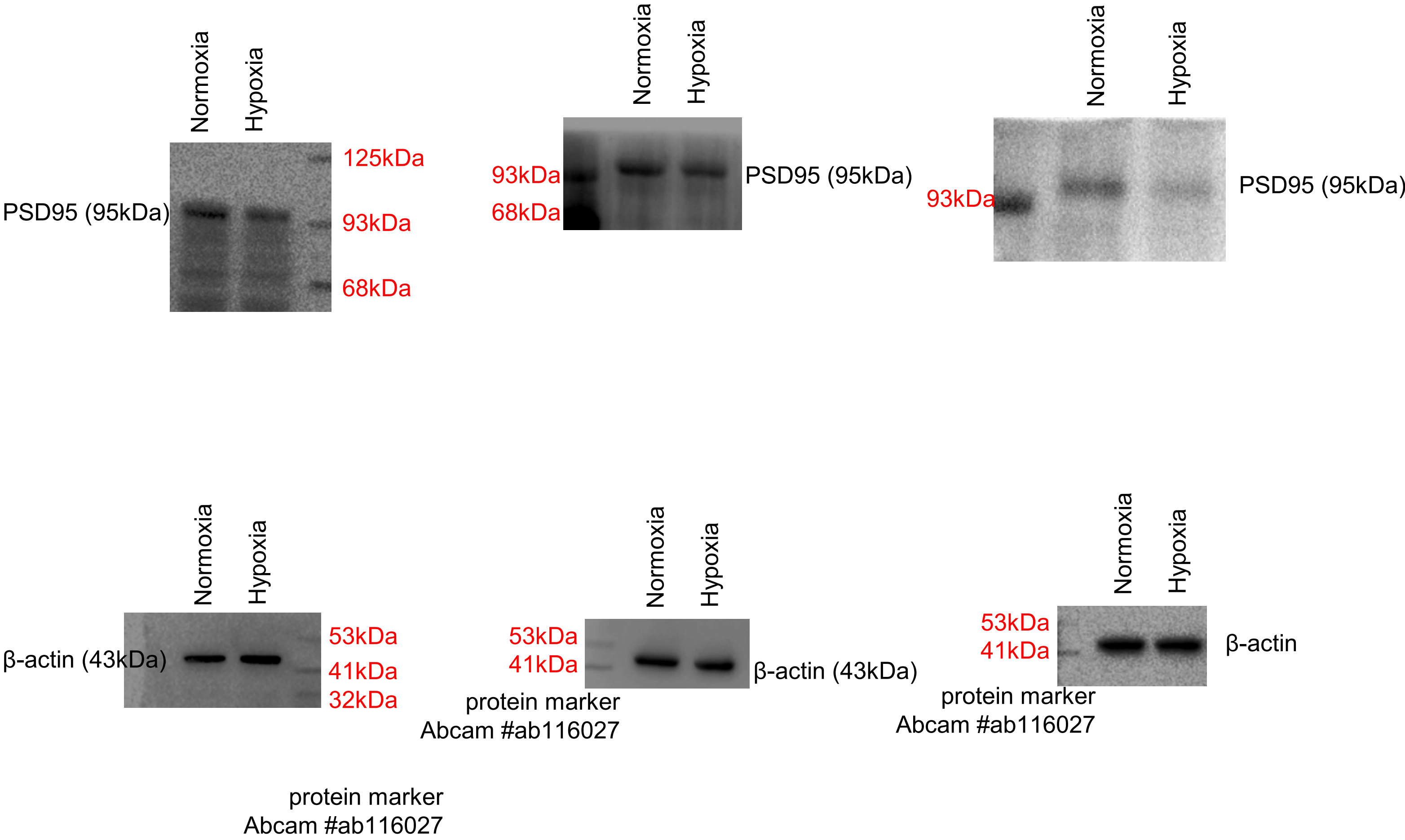

Figure 3E. HT-22 cells

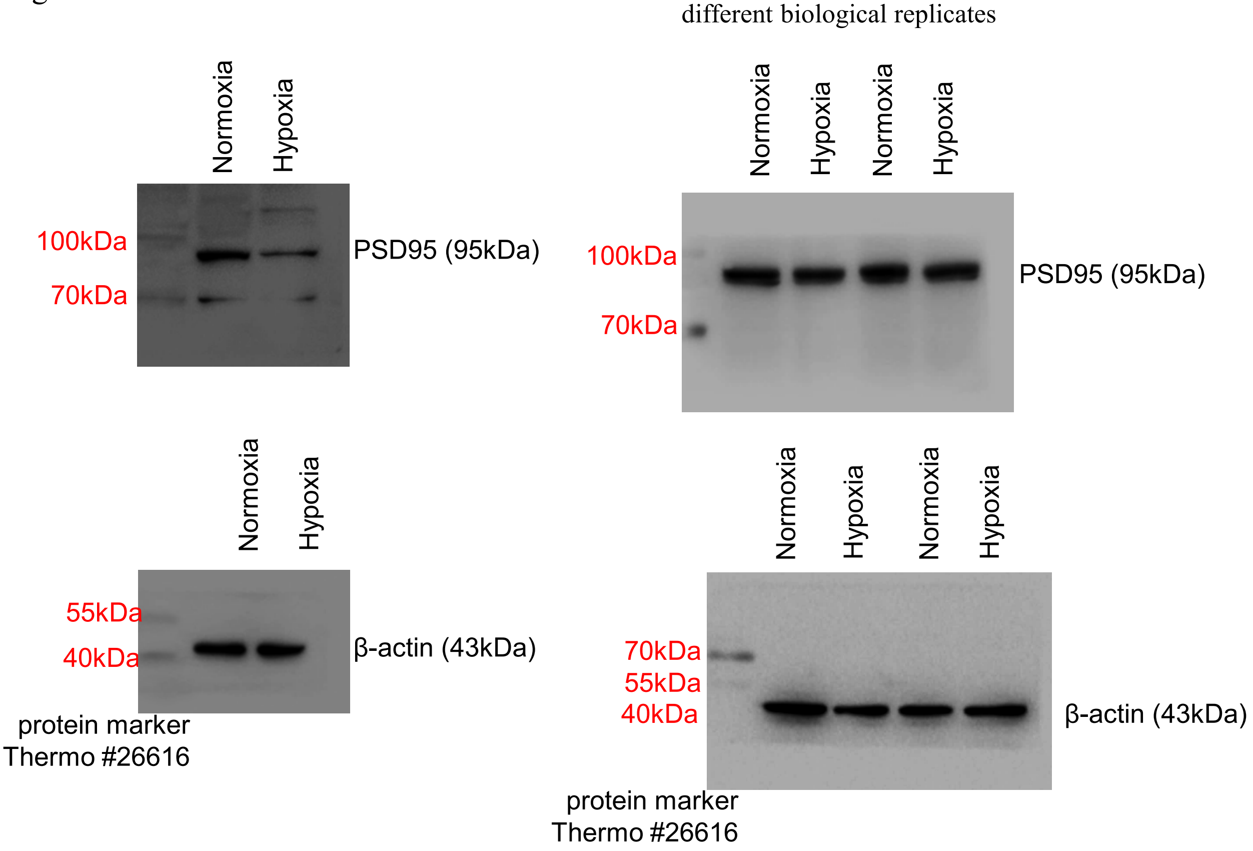

Figure 3G. HT-22 cells

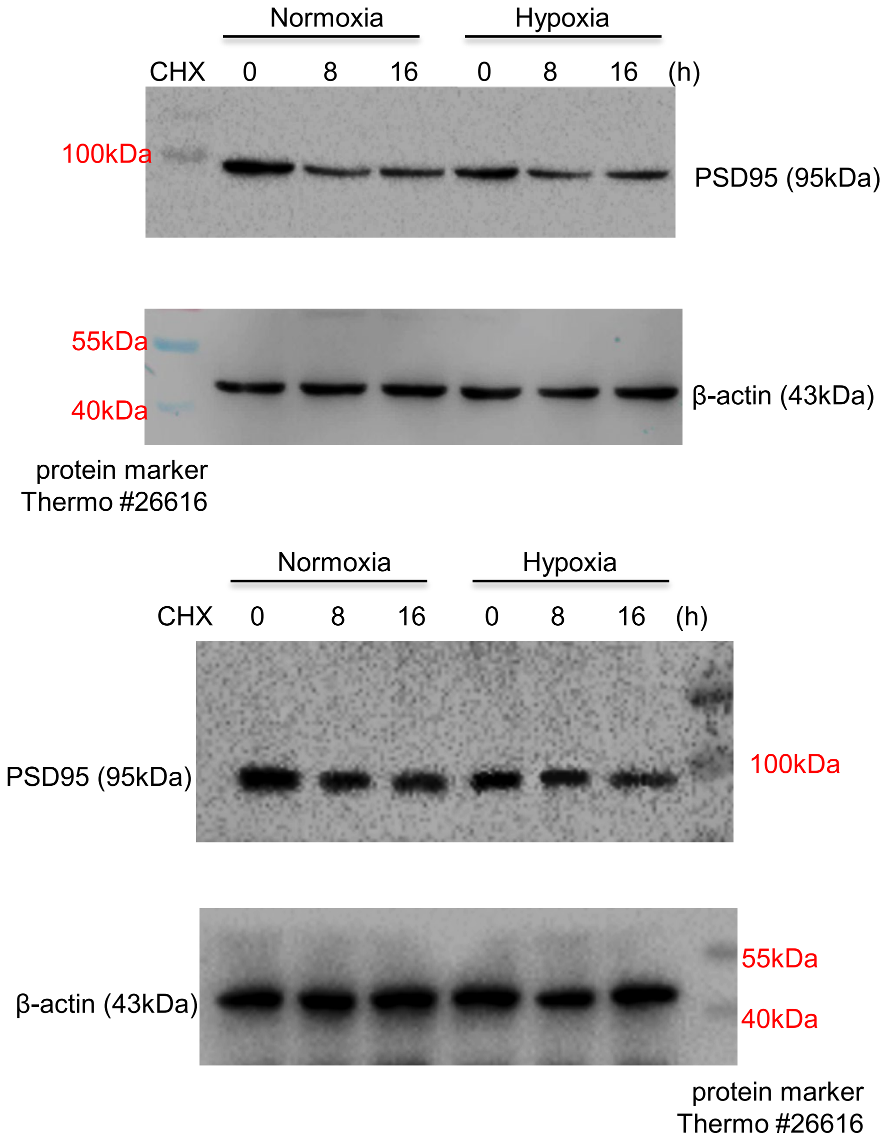

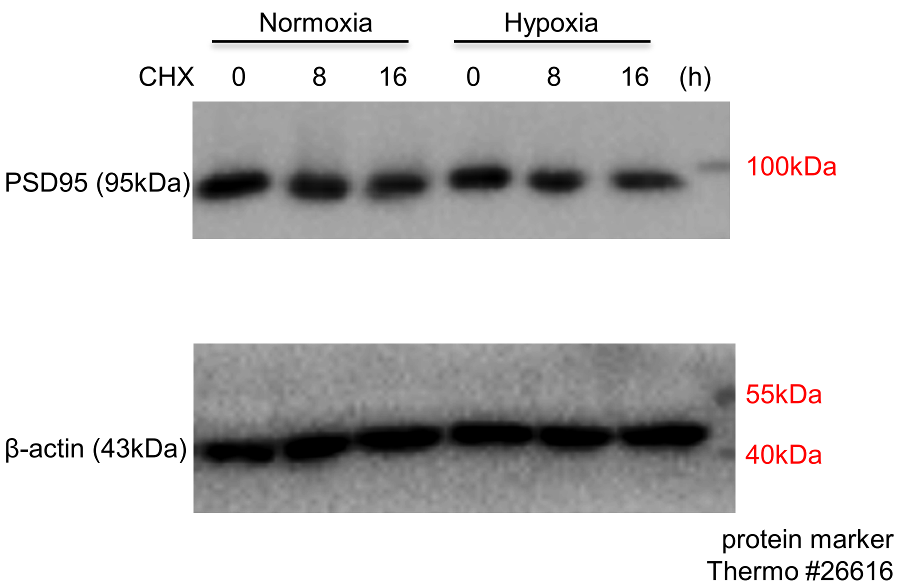

Figure 3J. Mouse hippocampus

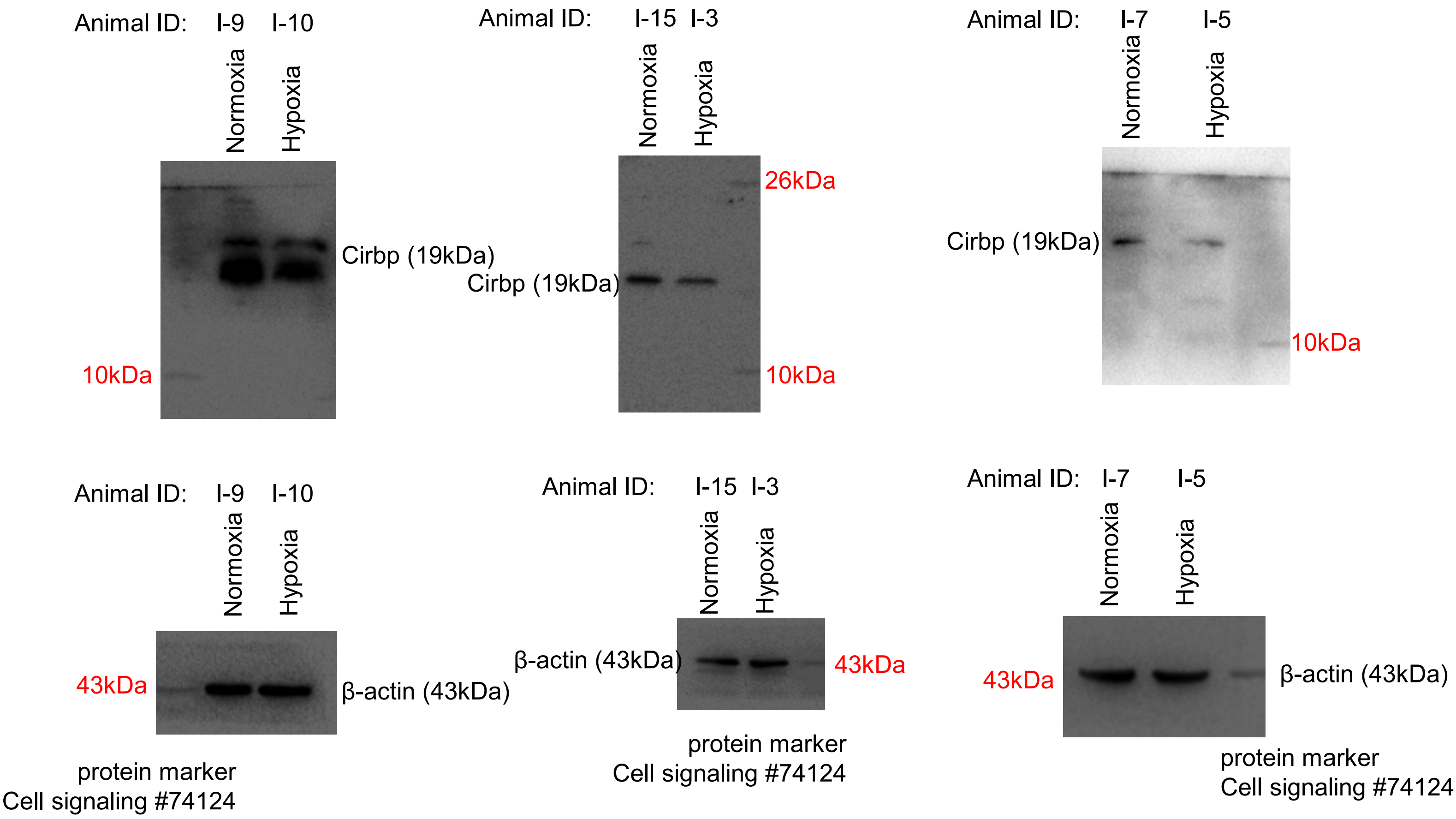

Figure 3K. Primary hippocampal neurons

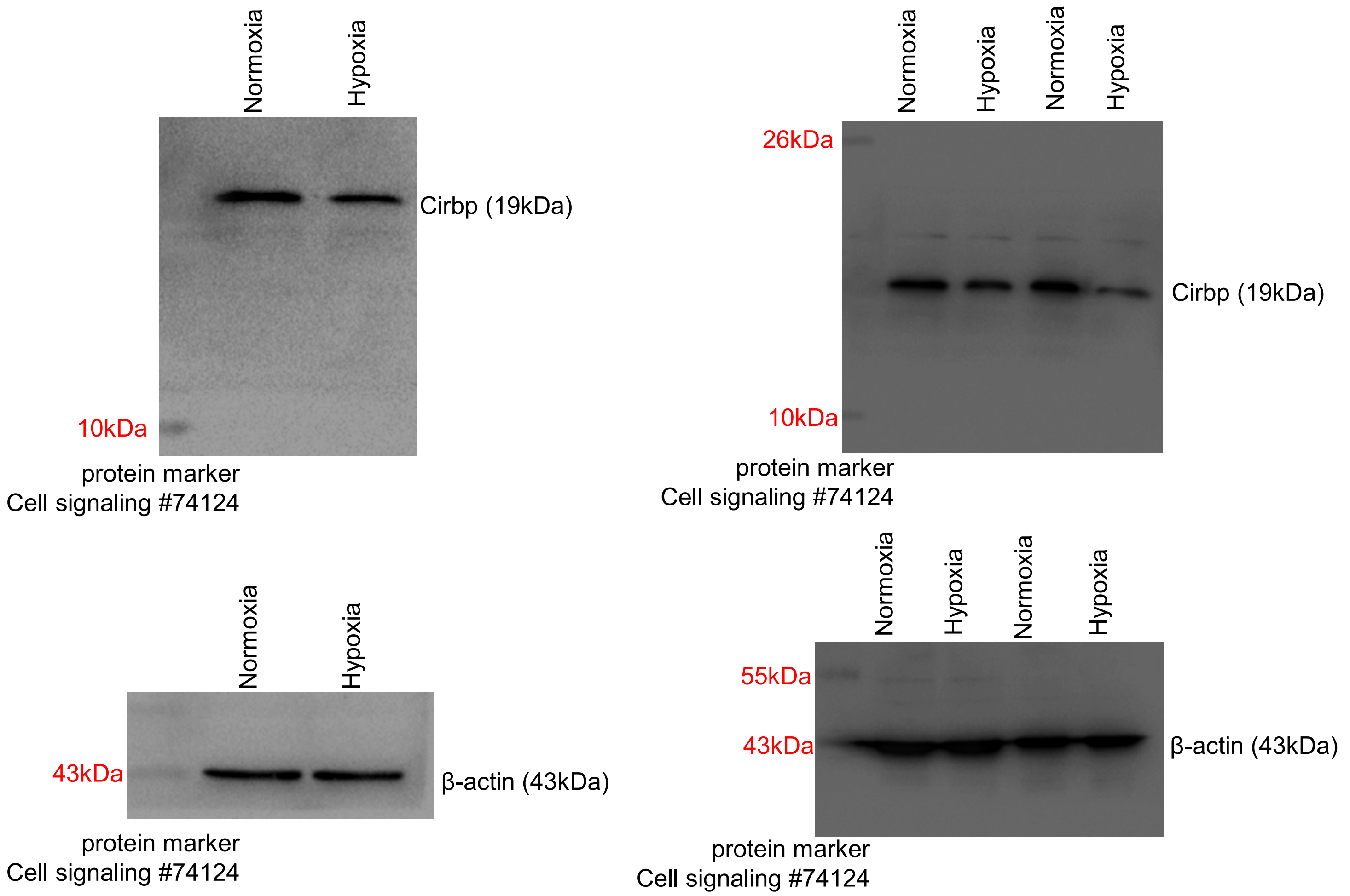

Figure 3L. HT-22 cells

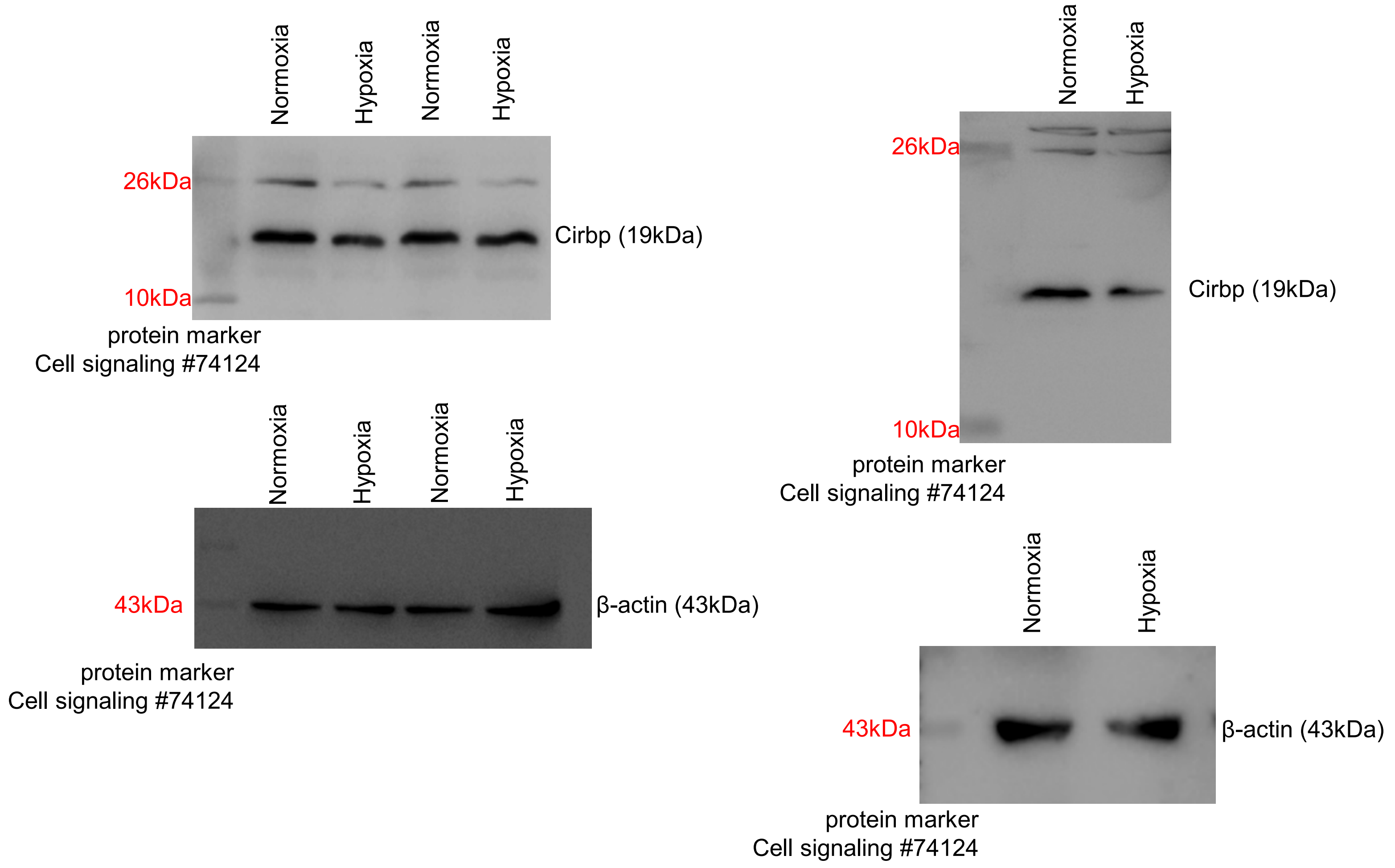

Figure 4A. Primary hippocampal neurons

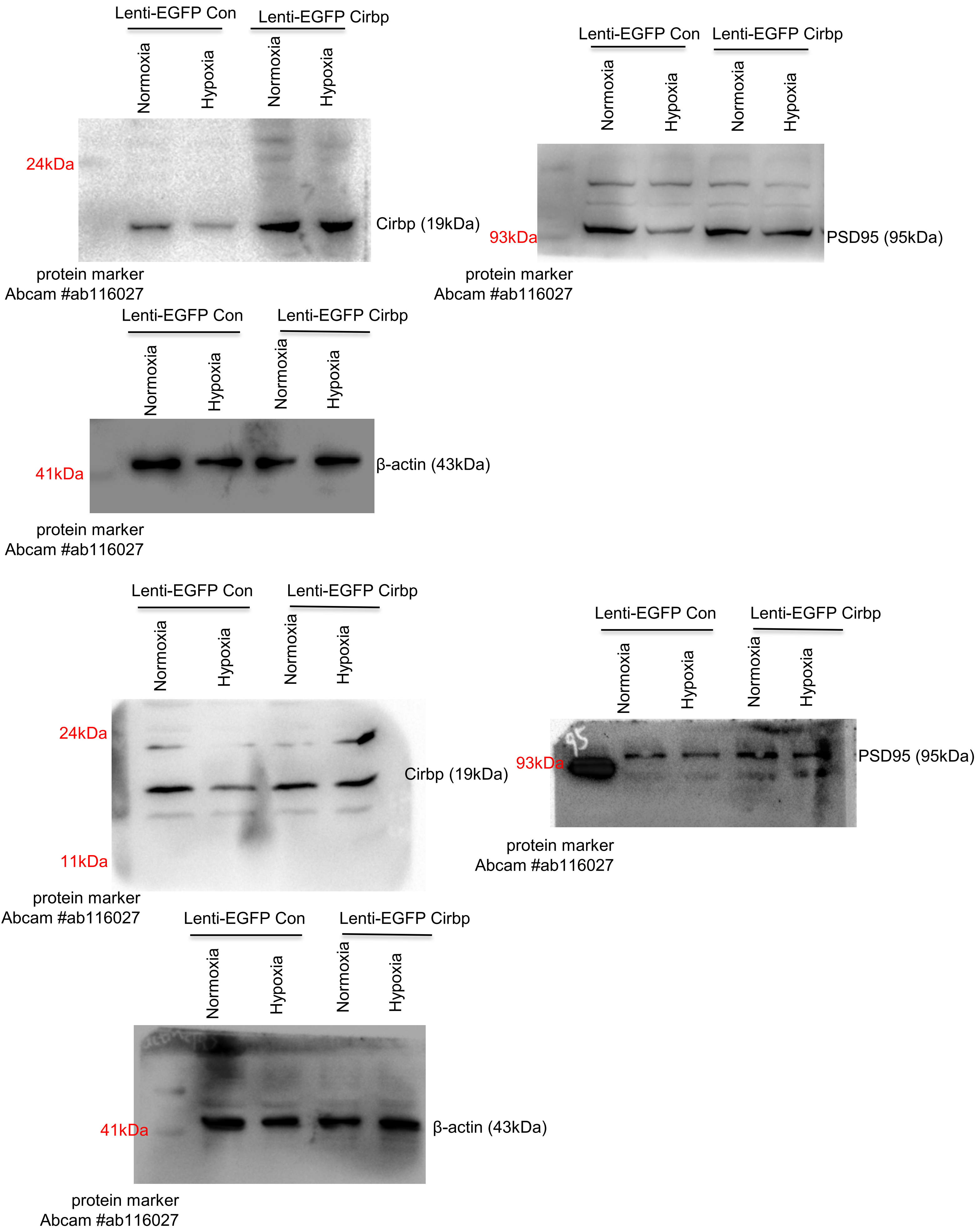

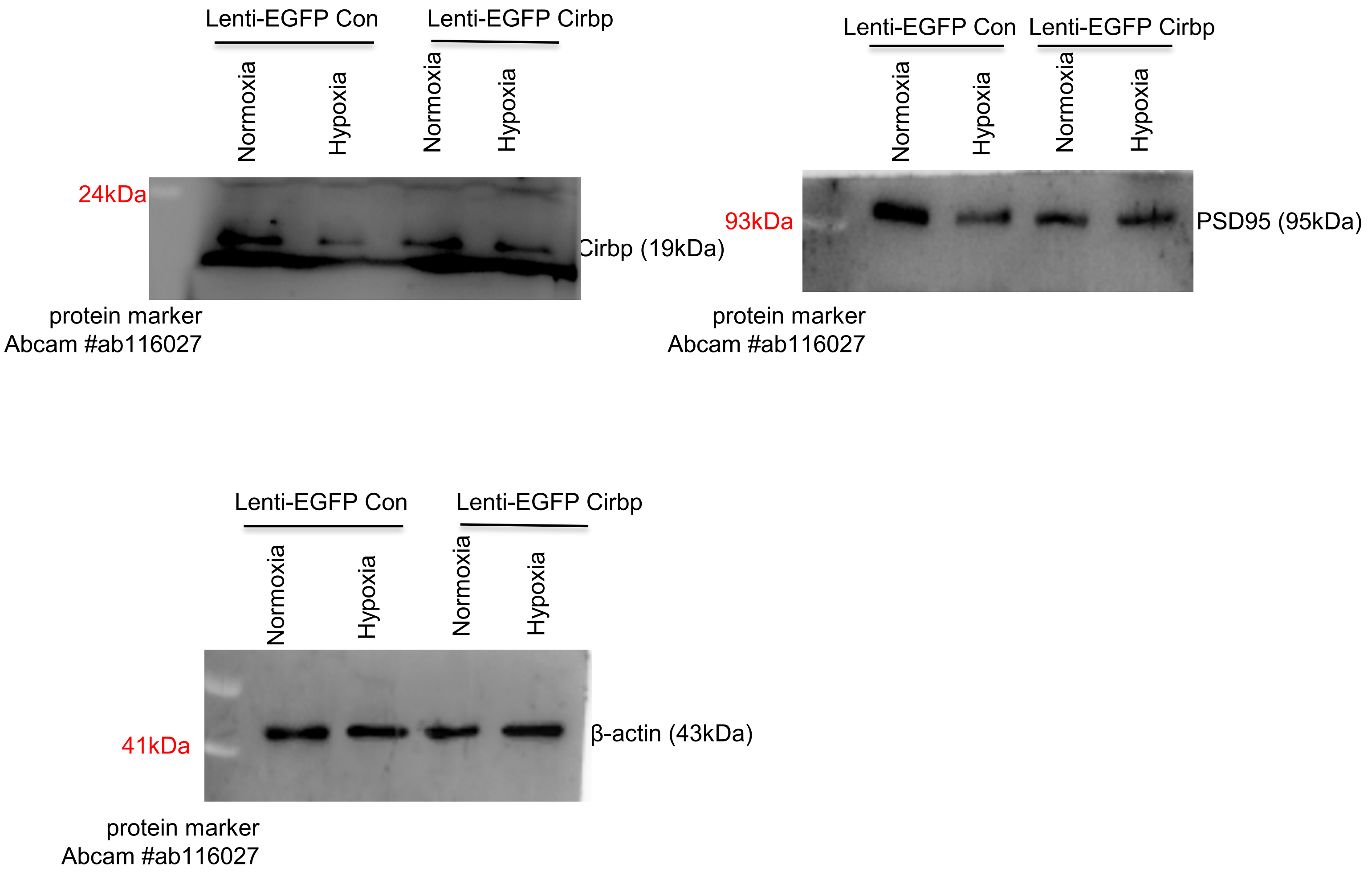

Figure 4B. HT-22 cells

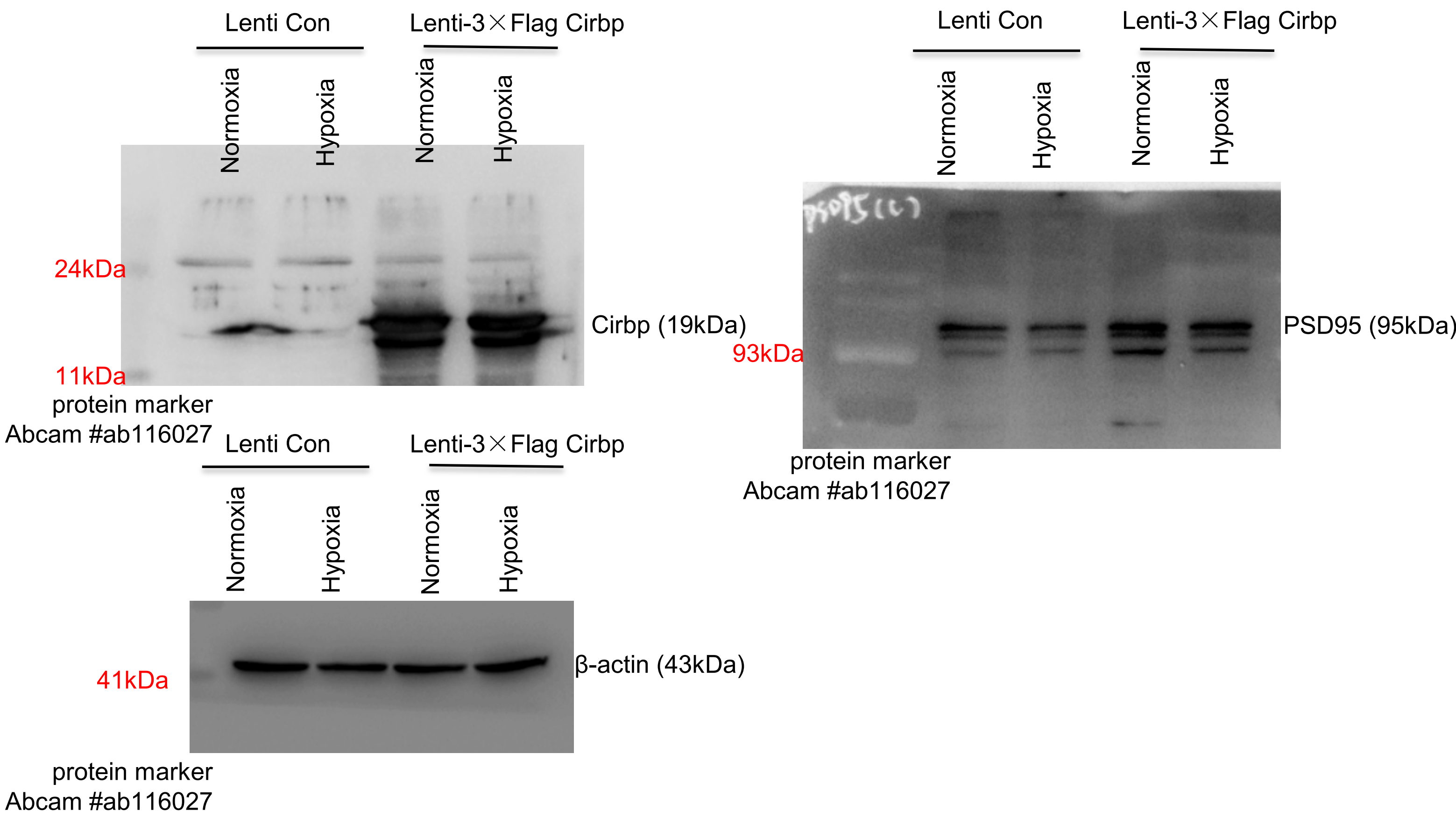

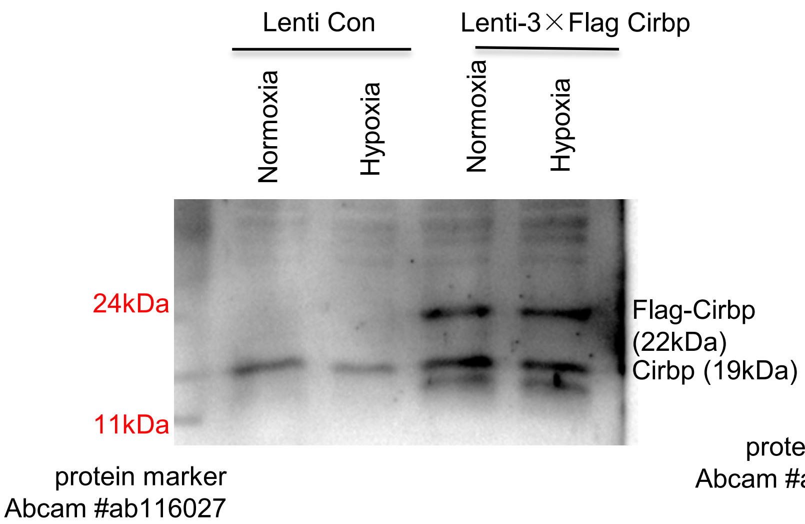
Lenti Con
Lenti-3×Flag Cirbp
Normoxia
Hypoxia
Normoxia
Hypoxia
24kDa
11kDa
Flag-Cirbp (22kDa)
Cirbp (19kDa)
protein marker
Abcam #ab116027

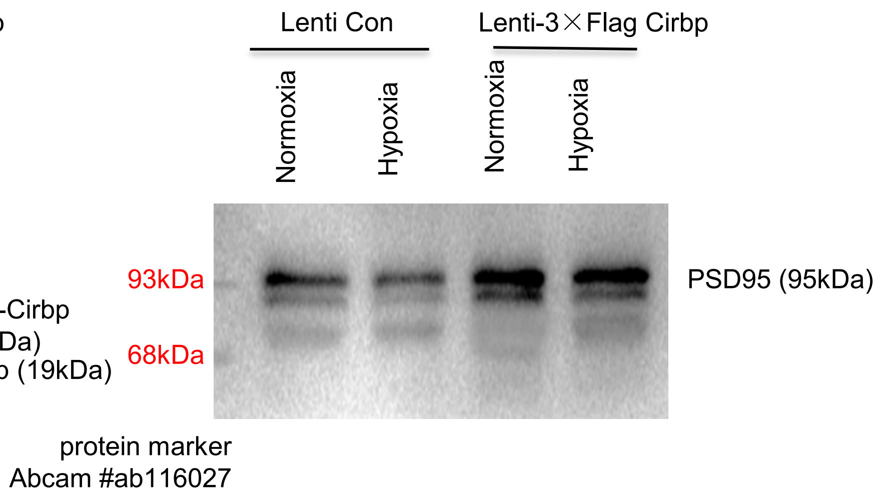
Lenti Con
Lenti-3×Flag Cirbp
Normoxia
Hypoxia
Normoxia
Hypoxia
93kDa
68kDa
PSD95 (95kDa)
protein marker
Abcam #ab116027

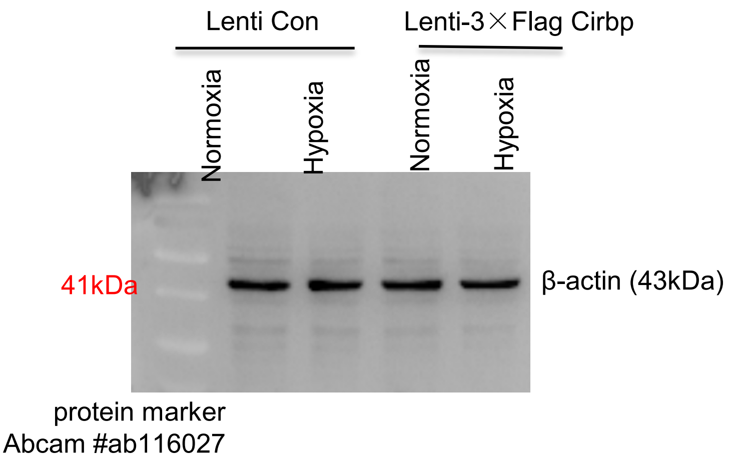
Lenti Con
Lenti-3×Flag Cirbp
Normoxia
Hypoxia
Normoxia
Hypoxia
41kDa
β-actin (43kDa)
protein marker
Abcam #ab116027

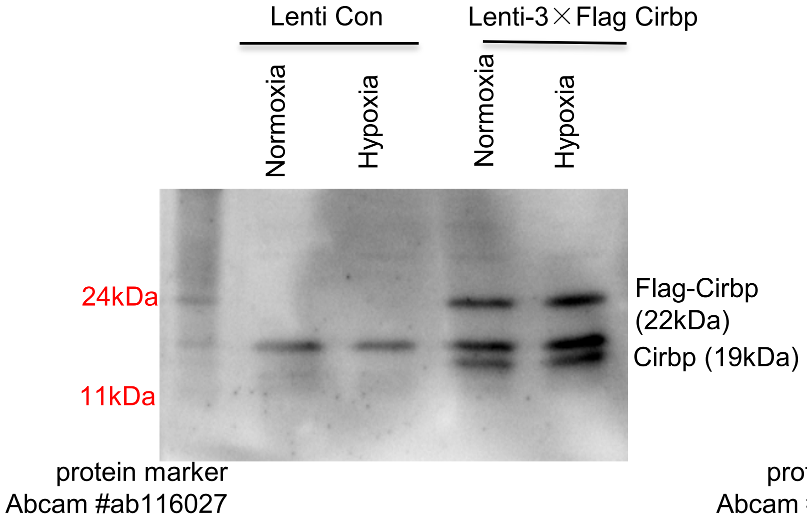
Lenti Con
Lenti-3×Flag Cirbp
Normoxia
Hypoxia
Normoxia
Hypoxia
24kDa
11kDa
Flag-Cirbp (22kDa)
Cirbp (19kDa)
protein marker
Abcam #ab116027

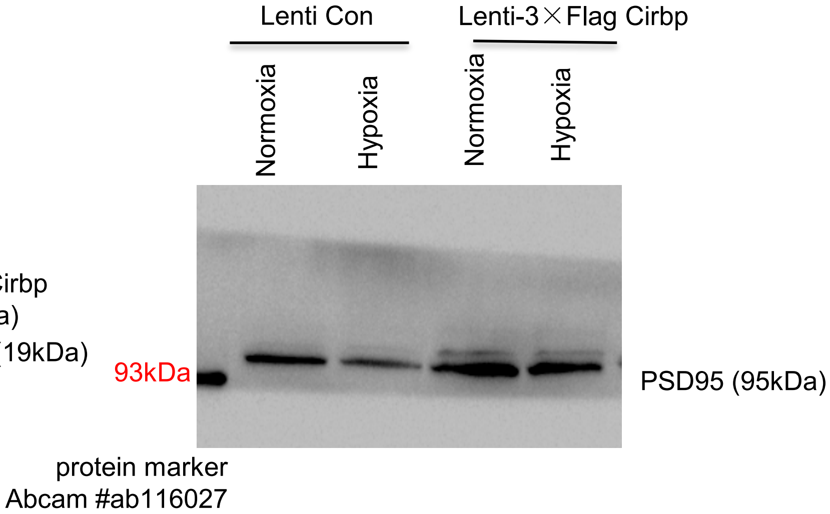
Lenti Con
Lenti-3×Flag Cirbp
Normoxia
Hypoxia
Normoxia
Hypoxia
93kDa
PSD95 (95kDa)
protein marker
Abcam #ab116027

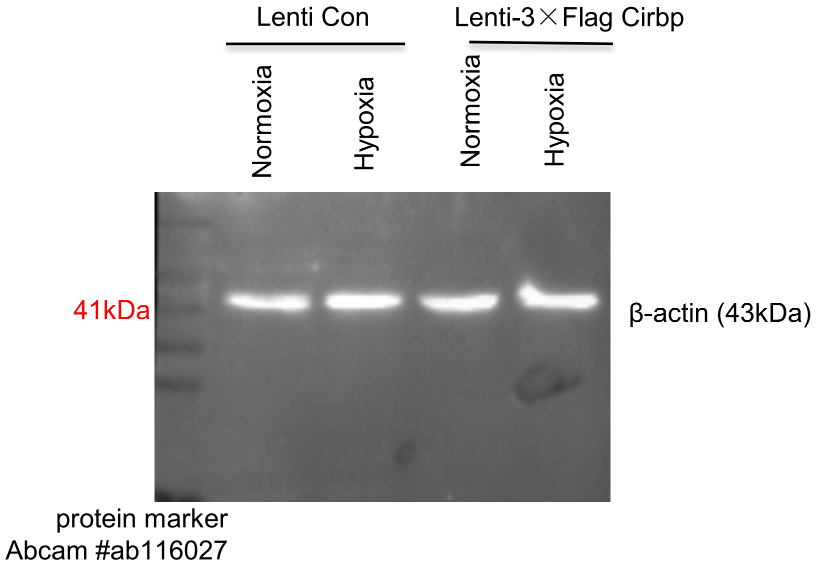
Lenti Con
Lenti-3×Flag Cirbp
Normoxia
Hypoxia
Normoxia
Hypoxia
41kDa
β-actin (43kDa)
protein marker
Abcam #ab116027

Figure 5B. Mouse hippocampus

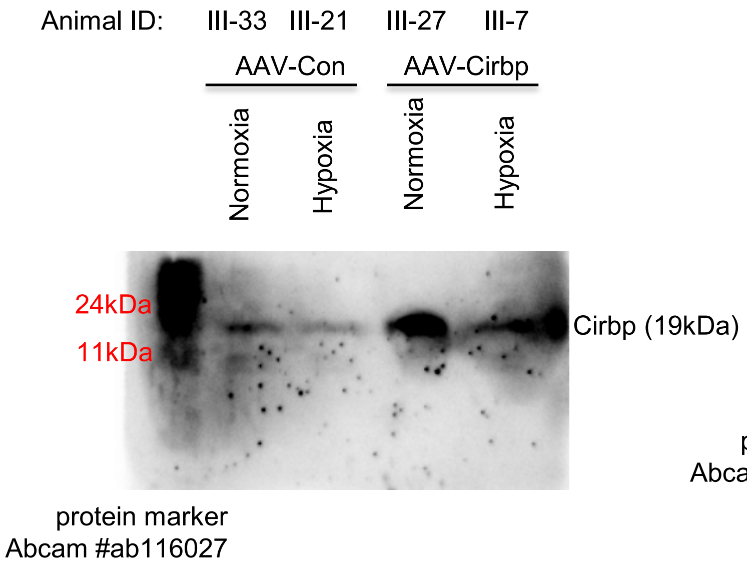

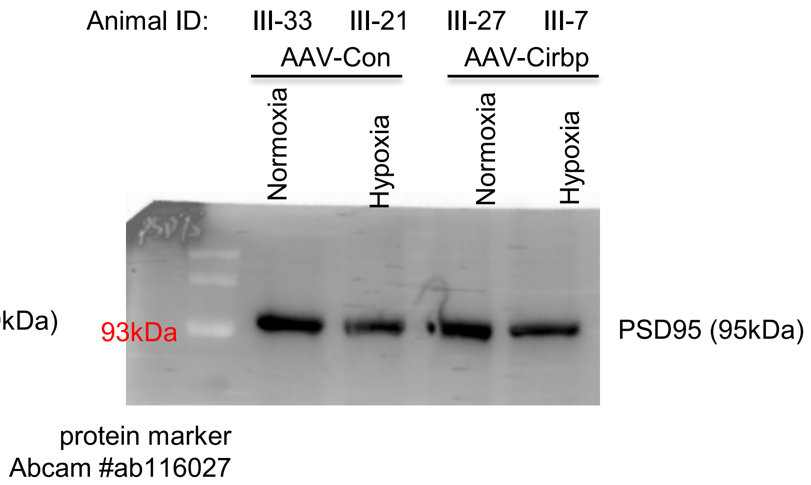

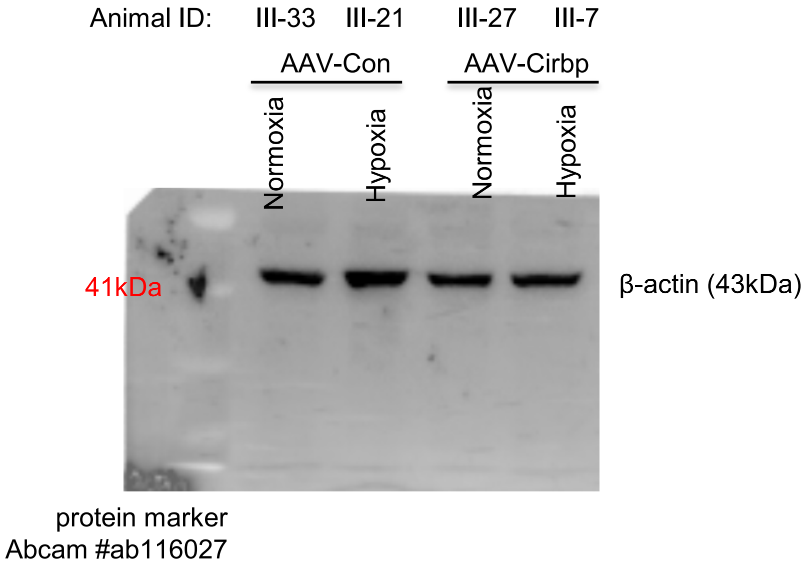

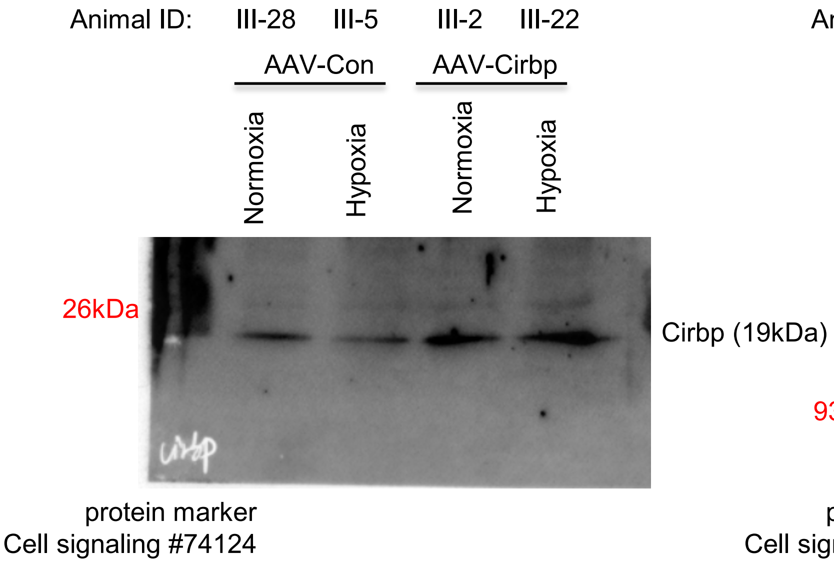
Animal ID:
III-28
III-5
III-2
III-22
AAV-Con
AAV-Cirbp
Normoxia
Hypoxia
Normoxia
Hypoxia
26kDa
Cirbp (19kDa)
protein marker
Cell signaling #74124

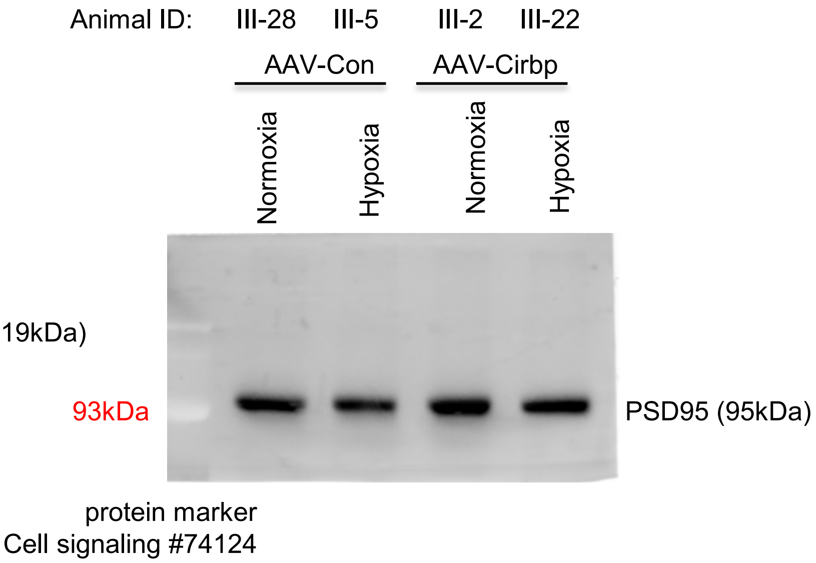
Animal ID:
III-28
III-5
III-2
III-22
AAV-Con
AAV-Cirbp
Normoxia
Hypoxia
Normoxia
Hypoxia
93kDa
PSD95 (95kDa)
protein marker
Cell signaling #74124

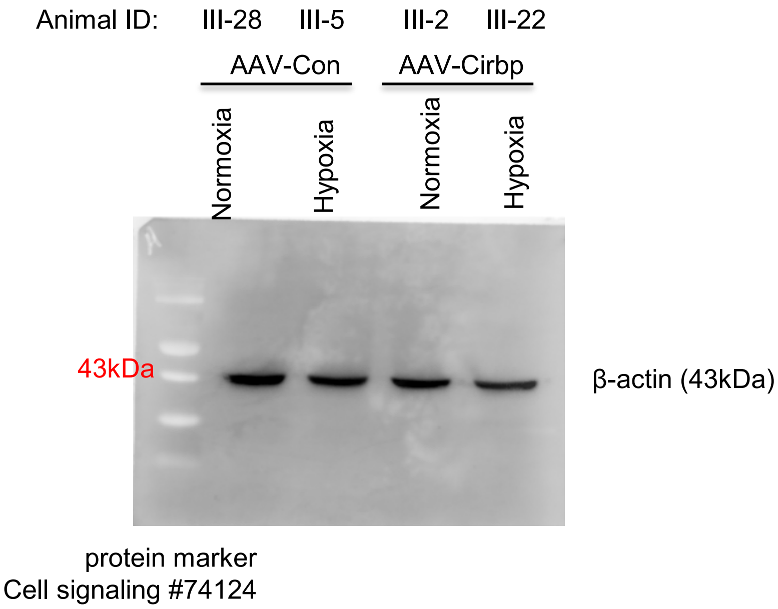
Animal ID:
III-28
III-5
III-2
III-22
AAV-Con
AAV-Cirbp
Normoxia
Hypoxia
Normoxia
Hypoxia
43kDa
β-actin (43kDa)
protein marker
Cell signaling #74124

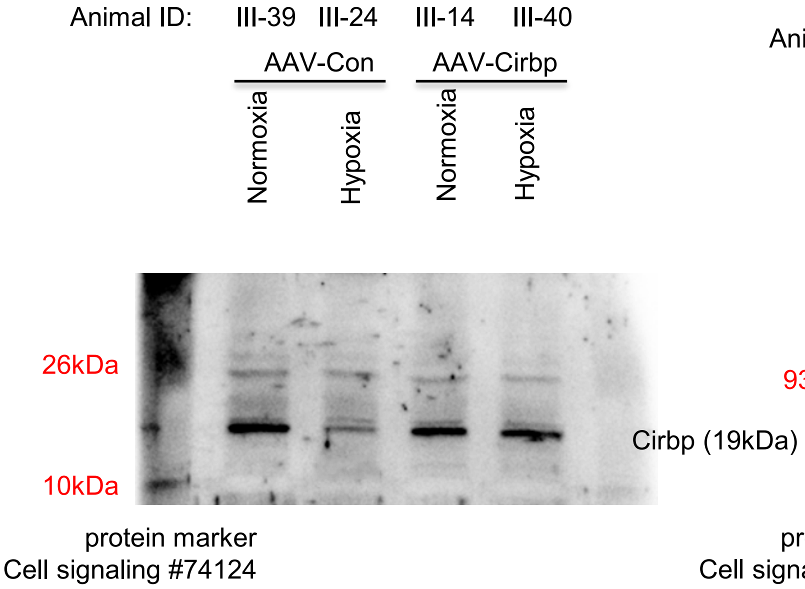
Animal ID:
III-39
III-24
III-14
III-40
AAV-Con
AAV-Cirbp
Normoxia
Hypoxia
Normoxia
Hypoxia
26kDa
10kDa
Cirbp (19kDa)
protein marker
Cell signaling #74124

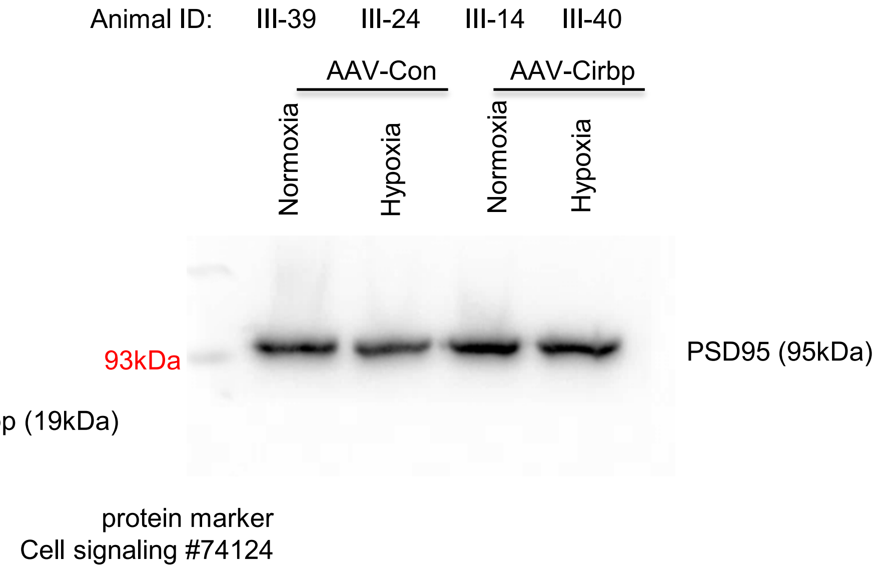
Animal ID:
III-39
III-24
III-14
III-40
AAV-Con
AAV-Cirbp
Normoxia
Hypoxia
Normoxia
Hypoxia
93kDa
PSD95 (95kDa)
protein marker
Cell signaling #74124

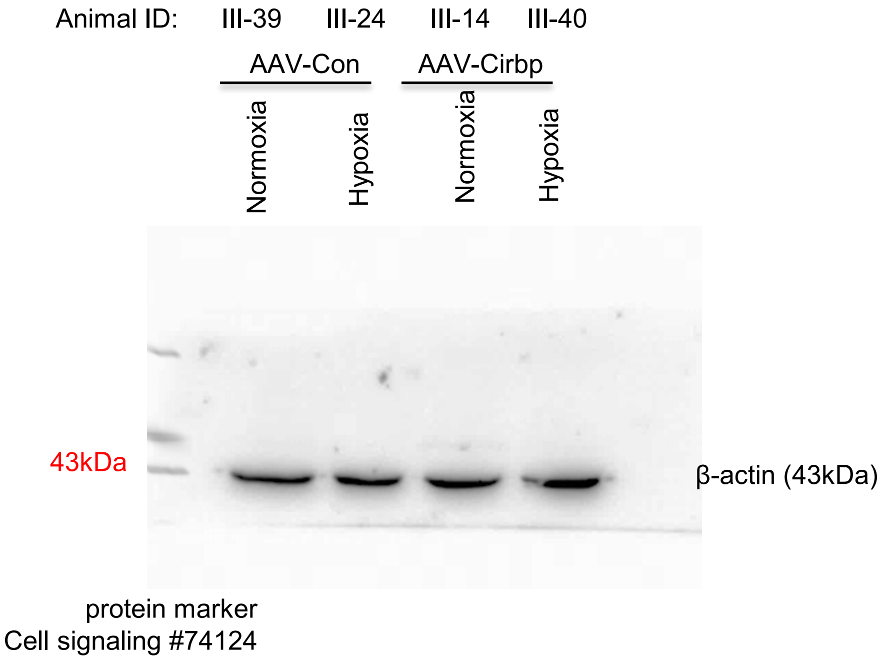
Animal ID:
III-39
III-24
III-14
III-40
AAV-Con
AAV-Cirbp
Normoxia
Hypoxia
Normoxia
Hypoxia
43kDa
β-actin (43kDa)
protein marker
Cell signaling #74124
